# Membraneless protocell confined by a heat flow

**DOI:** 10.1038/s41567-025-02935-4

**Published:** 2025-06-26

**Authors:** Alexander Floroni, Noël Yeh Martín, Thomas Matreux, Laura I. Weise, Sheref S. Mansy, Hannes Mutschler, Christof B. Mast, Dieter Braun

**Affiliations:** 1 Systems Biophysics, Ludwig Maximilian University Munich, Munich, Germany; 2 Institute of Biotechnology HiLIFE, Helsinki Institute of Life Sciences, University of Helsinki, Helsinki, Finland; 3 Max Planck Institute of Biochemistry, Planegg, Germany; 4 Department of Cellular, Computational and Integrative Biology, University of Trento, Trento, Italy; 5 Department of Chemistry, University of Alberta, Edmonton, Alberta Canada; 6 Department of Chemistry and Chemical Biology, TU Dortmund University, Dortmund, Germany

**Keywords:** Complex networks, Biological physics, Permeation and transport

## Abstract

In living cells, a complex mixture of biomolecules is assembled within and across membranes. This non-equilibrium state is maintained by sophisticated protein machinery, which imports food molecules, removes waste products and orchestrates cell division. However, it remains unclear how this complex cellular machinery emerged and evolved. Here we show how the molecular contents of a cell can be coupled in a coordinated way to non-equilibrium heat flow. A temperature difference across a water-filled pore assembled the core components of a modern cell, which could then activate the gene expression. The mechanism arose from the interplay of convection and thermophoresis, both driven by the same heat source. The cellular machinery of protein synthesis from DNA via RNA was triggered as a direct result of the concentration of cell components. The same non-equilibrium setting continued to attract food molecules from an adjacent fluid stream, keeping the cellular molecules in a confined pocket protected against diffusion. Our results show how a simple non-equilibrium physical process can assemble the many different molecules of a cell and trigger its basic functions. The framework provides a membrane-free environment to bridge the long evolutionary times from an RNA world to a protein-based cell-like proto-metabolism.

## Abstract (web summary)

It is unclear how cell compartmentalization emerged in prebiotic conditions. Now it is shown that a temperature gradient in a confined space can bring the core components of a cell together.

## Main

The assembly of a living cell by piecing together the different molecular components is a formidable puzzle and the ultimate target of synthetic biology worldwide^1–6^. However, unlike living cells, which form and inherit the cellular chassis from their progenitors, the de novo generation of a cell-like compartment with similar properties is non-trivial. In particular, it is difficult to imagine how a dense and functional cytosol could be generated from a dispersed pool of molecules without a complex cell membrane and membrane proteins that provide homeostatic conditions for cellular self-assembly and self-sustainment. This interdependence resembles a classic chicken-and-egg problem: how to synthesize and evolve membrane proteins if initial membranes cannot provide the accumulation and feeding mechanisms needed to make the membrane proteins in the first place?

Despite the difficulty, the construction of systems that mimic biological cells shows substantial progress, also providing a better understanding of cellular structures and biological mechanisms. A multitude of cellular mechanisms and behaviours have been reconstituted within synthetic membranes including fundamental ones like transcription, translation and replication^7–12^. Methods that reconstitute cellular membranes comprising embedded transport proteins and providing other cellular functions have improved considerably. However, none was successful at providing a fully functional cell membrane that forms autonomously in prebiotic conditions and self-regulates without outside intervention^13^. Therefore, passive transport and other strategies were necessary to control and manipulate the molecular concentration within cell-like compartments^14–21^. Some of these strategies rely on controlling the compartment properties by osmotic pressure^14,15^, whereas others exploit temperature oscillations^17^, light^21^, diffusiophoresis^18^ or pH^19,20^. Furthermore, alternative encapsulation methods have been proposed, the most prebiotically relevant being coacervation. In this case, the coacervates assume the role of the membrane to protect the PURExpress (hereafter referred to as TX-TL) reaction from dilution^22,23^. Although progress has been made in this direction, the compatibility of coacervates with complex reaction networks remains a challenge^23^.

Substantial progress has also been made in the conception of systems that enable the continuous feeding of cell-like reactions. Pump-based, highly structured microfluidic compartments^24,25^, with surface-bound DNA^26^ or separated by a dialysis membrane^27^, are highly relevant in this regard. Hydrogels that bind molecular components^28^ provide another powerful alternative contributing to this array of vesicle-free platforms to control and host protein synthesis under serial dilution and feeding. Furthermore, numerous advancements were made in the communication between artificial cells^29^ and between artificial and biological cells^30,31^, which requires a certain degree of selective membrane permeability.

In this work, we show that simple heat-flow-traversed, water-filled channels constitute an open system, which requires minimal outside intervention and is compatible with prebiotic conditions. This simple non-equilibrium setting achieves cell-like organization by concentrating and confining the purified molecules of a cell. As a proof of principle, we used this mechanism to accumulate a complex, dispersed and inactive mixture of molecules into a functioning cell-like assembly. In this conception of a ‘thermally assembled protocell’, the region that hosts the accumulated material functions as an active mimic of a cytosol confined by an easy-to-traverse temperature difference rather than a physical barrier.

Temperature differences at the microscale have been shown to selectively accumulate molecules by driving their thermophoresis and fluid convection^32–35^. Thermophoresis typically moves molecules to the cold side, whereas moderate laminar thermal convection flows amplify the accumulation downwards. This non-equilibrium setting has been shown to enhance ribozyme activity by divalent salt accumulation^33^ or the generation and maintenance of pH gradients without semipermeable membranes^34^. The shown accumulation mechanism has been the most effective for slow-diffusing DNA, especially for non-physiological low salt concentrations^32,36–38^. It was, thus, not clear whether the accumulation at physiological salt concentrations and temperatures would be sufficient to equally accumulate such a diverse group of molecules, consisting of ions, ribonucleotides, DNA, RNA, amino acids, proteins and ribosomes, namely, the essential molecular building blocks of modern cells.

Previously, thermal non-equilibrium settings at air–water interfaces used a different mechanism of evaporation-based capillary flow^39,40^ to drive molecular evolution through replication and strand separation at low temperatures, suggesting thermal gradients for the early RNA world. Therefore, the shown experiments offer a thermal bridge from early RNA evolution to complex cellular assemblies.

## Results

**Fig. 1:**
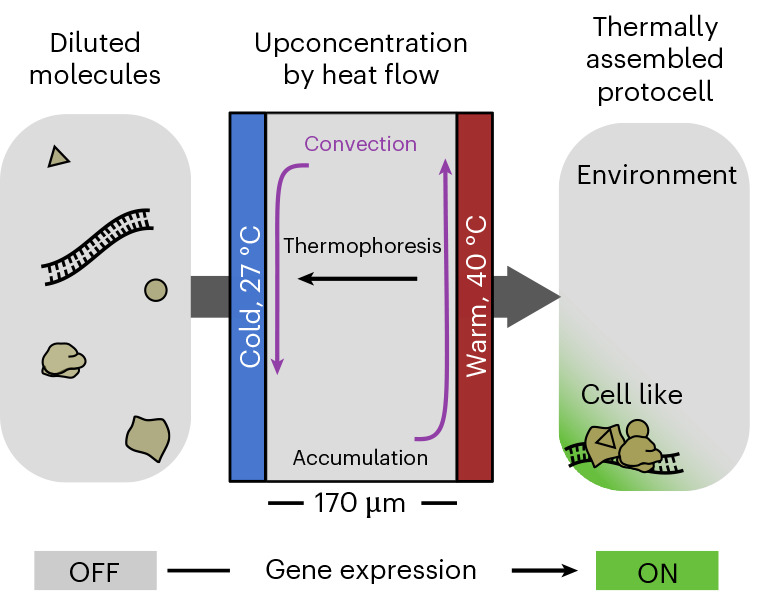
Thermally assembled protocell. A microscale reaction chamber was filled with the components of a recombinant in vitro transcription–translation system at a concentration that was too low to support biological activity. The application of a temperature difference triggered thermophoresis and convection inside the solution. The combination of both effects concentrated the TX-TL components towards the bottom of the chamber and, thus, activated the gene expression (green area).

In the following, we used heat flows to accumulate and confine a functioning cytoplasmic mimic from a concentration of components that was too low to support activity (Fig. 1). To that end, we used TX-TL (PURExpress) as a model of a minimal cytoplasm of a living cell. PURExpress is a commercially available version of the PURE system^41^ and consists of the purified *Escherichia coli* translation machinery, T7 RNA polymerase, an adenosine triphosphate (ATP) regeneration system, ribonucleotide tri-phosphates, transfer RNAs (tRNAs), amino acids, salts and cofactors.

We developed custom-made chambers that allowed the establishment of a well-controlled temperature difference across a gap of 170 µm and permitted optical access for real-time fluorescence imaging under conditions optimized for low protein adsorption^33,34^ (Supplementary Figs. 1 and 2). To confirm the compatibility of the setup with cell-free protein synthesis, we supplemented the TX-TL solution with a linear DNA template encoding superfolder green fluorescent protein (sfGFP)^42^. As expected, we observed a homogeneous, time-dependent increase in green fluorescence over the whole chamber on incubation at 37 °C (Supplementary Fig. 3).

### Thermal accumulation as the basis for protocell assembly

After having confirmed the compatibility of TX-TL with the setup of the thermophoretic chamber, we next sought to explore the accumulation behaviour of the system within an externally applied thermal gradient. Two processes dominate the motion of solute molecules on the application of a temperature difference at the microscale. First, molecules consistently move along the temperature differences in our settings from the warm to the cold side. This effect is termed thermophoresis or thermal diffusion. Second, the density difference in the differentially heated solution creates a laminar flow, which shuttles the fluid downwards on the denser, colder side. At moderate convection speeds, solute accumulation is enhanced by the superposition of both effects. The combined effect is termed thermogravitational or thermal accumulation and has been recently experimentally and computationally demonstrated to be capable of accumulating biomolecules in simple molecular solutions^32,34,37,40^.

To characterize the thermophoretic properties of our reporter molecule sfGFP in the viscous environment of the TX-TL solution, we initially conducted a numerical model using finite element methods (the ‘Material and methods’ section in Supplementary Information). The model accounted for heat conduction, laminar fluid dynamics, gravity, thermophoresis and diffusion. Using this model, we found that a chamber thickness of 170 µm leads to an optimal convection speed that maximizes the accumulation of sfGFP within our experimental timescale (Supplementary Fig. 4a). The experimental data fitted best to a model in which the viscosity of the TX-TL solution was 1.3-fold higher than water (see the ‘Material and methods’ section in Supplementary Information).

**Fig. 2:**
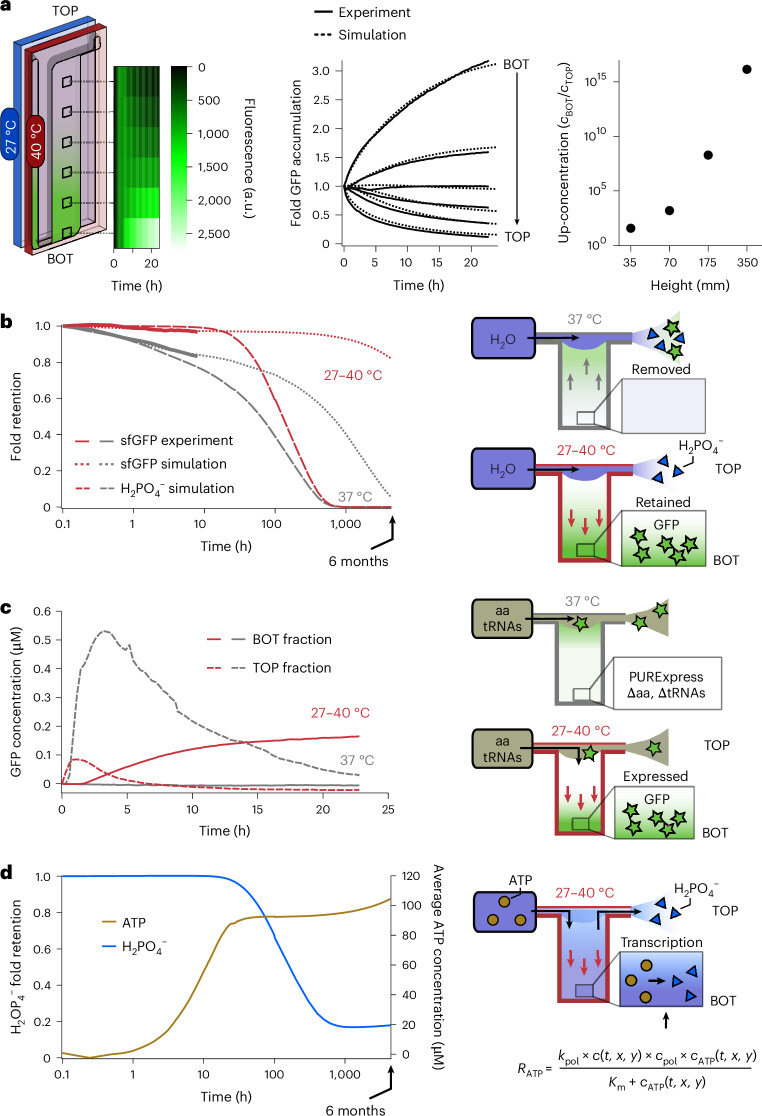
Compartmentalization and feeding by a temperature difference. **a**, A thermophoretic chamber filled with a TX-TL solution, expressing sfGFP at the steady state, was subjected to a temperature difference (27–40 °C). The accumulation of sfGFP was monitored by measuring fluorescence over time at different chamber positions (black squares), recording a 3-fold absolute and 25-fold relative accumulation (solid lines). The dashed lines correspond to a simulated finite element model (see the ‘Material and methods’ section in Supplementary Information). **b**, The setting was challenged by a continuous flow of water at the top of the chamber (11 µl h^–1^; dark blue) to test the thermal confinement of sfGFP provided by the temperature difference. Without the temperature difference, diffusion into the outward flow slowly removed sfGFP from the chamber (grey arrows). The total pre-expressed sfGFP fluorescence remaining inside the chamber was modelled by finite element simulations for up to 6 months (dotted lines). The solid lines are averages of three experiments. As a comparison, ${{\rm{H}}}_{2}{{\rm{PO}}}_{4}^{-}$ was simulated as a possible waste molecule produced during sfGFP synthesis (large, dotted lines). ${{\rm{H}}}_{2}{{\rm{PO}}}_{4}^{-}$ was removed much faster from the thermal trap than sfGFP under isothermal conditions but also when a temperature gradient was present. **c**, Activation and retention of a deficient TX-TL reaction was probed by supplying the missing amino acids and tRNAs at a flow rate of 11 µl h^–1^ at the top of the chamber. The sfGFP concentration was measured at the top and bottom of the chamber with and without applying a temperature difference. In isothermal conditions, the TX-TL reaction was first activated and then flushed out, leaving no synthesized sfGFP in the compartment in the long run. However, when a temperature difference was applied, the reaction was activated by the accumulated supply solution, leading to the synthesis and long-term accumulation and retention of sfGFP. **d**, Simulated kinetics of ATP (brown) and ${{\rm{H}}}_{2}{{\rm{PO}}}_{4}^{-}$ (blue) as feeding and waste molecules, respectively, in a simulated transcription reaction under an active thermal gradient. ATP was introduced in the trap at a concentration of 1 mM under the same fluid flow as in previous experiments and simulations. ${{\rm{H}}}_{2}{{\rm{PO}}}_{4}^{-}$, which is present in the trap before the start of the flow, is simultaneously removed from the trap by the continuous fluid flow at the top of the chamber and produced from ATP throughout the trap corresponding to the concentration of the TX-TL reaction. A stable state is reached after 800 h, in which ${{\rm{H}}}_{2}{{\rm{PO}}}_{4}^{-}$ is produced at the same rate as it is removed. On the other hand, ATP is continuously accumulating in the chamber and retained against the fluid flow.

Next, we ascertained whether the thermophoretic accumulation of sfGFP was possible within the highly complex molecular mixture of a TX-TL system. We initially expressed sfGFP at 37 °C for 4 h until reaching the plateau phase and then applied a temperature difference between 27 °C and 40 °C. This triggered the accumulation of sfGFP, which reached an accumulation steady state after about 24 h (Supplementary Fig. 5a and Fig. 2a (solid lines)). Fluorescence microscopy revealed an ~3-fold sfGFP accumulation at the bottom of the chamber with respect to the fluorescence level obtained at the plateau phase of protein synthesis before applying the temperature difference. The fluorescence at the top was instead depleted (solid lines). The experimental fluorescence data were fitted by the finite element model to estimate the thermophoretic mobility of sfGFP within the complex TX-TL solution (Fig. 2a (dotted lines); see the ‘Material and methods’ section in Supplementary Information).

Importantly, we found similar sfGFP fluorescence levels when the temperature difference was applied from the start of the experiment (Supplementary Fig. 5b,c), indicating simultaneous accumulation and gene expression. The maximum temperature difference that we could use for the accumulation experiments was found by the isothermal expression of sfGFP in a test tube. sfGFP was expressed from 27 °C to 42 °C, showing a maximum protein yield in the range of 33–39 °C (Supplementary Fig. 6), in agreement with a previous report^25^.

These results indicated that even without an active membrane compartment, two spatially distinct regions formed within the differentially heated chamber, consisting of a depleted region at the top and a region at the bottom that was enriched in sfGFP. We, therefore, hypothesized that the upper region could form an extracellular space, whereas the bottom region could give rise to a confined, functional mimic of a crowded cytoplasm.

### Thermally induced confinement

The cytoplasm of a cell is confined and separated from the environment by a semipermeable membrane. Cell membranes control the exchange of molecules with their surroundings and help tolerate different stress conditions. For example, endothelial cells regulate the molecular exchange between the bloodstream and neighbouring tissues. They do so without being disrupted or diluted by the blood flow and actively maintain their cytosolic components at working concentrations^43^.

To determine if a temperature difference could give rise to similar properties, we opened the top part of the chamber and provided a constant inflow and outflow of water at a rate of 11 µl h^–1^, 22 µl h^–1^ and 36 µl h^–1^ (Fig. 2b and Supplementary Fig. 7). In this way, we could assess whether the expressed and accumulated sfGFP could be confined and protected against dilution through a temperature difference rather than a semipermeable membrane.

To this end, TX-TL solutions were incubated with or without a temperature difference over 16 h to express and accumulate or only express sfGFP, respectively. Subsequently, the chambers were exposed to a flow of water at a rate of 11 µl h^–1^ for 9 h. A numerical model (Supplementary Fig. 8 and the ‘Material and methods’ section in Supplementary Information) predicted a near-complete removal of sfGFP after 6 months for the chamber incubated isothermally, in agreement with the experimentally recorded initial trend (Fig. 2b, grey lines). This result was expected by diffusion from the geometry features of the chamber with a height of 35 mm. Conversely, for the chamber subjected to a temperature difference, the model predicted a 97% retention of the sfGFP molecules after 9 h and still 83% after 6 months (Fig. 2b, red lines), even when the top of the chamber was continuously diluted by the water flow. The removal of small molecules in the chamber was simulated under the same conditions using orthophosphate (${{\rm{H}}}_{2}{{\rm{PO}}}_{4}^{-}$) as a waste molecule^44^ since the diffusion and Soret coefficients were known (959 µm^2^ s^–1^ and 0.7 × 10^–3^ K^–1^ (ref. ^34^), respectively). Phosphate is a byproduct of many processes in the TX-TL reaction including RNA polymerization. The inorganic phosphate competes with energy molecules such as ATP and guanosine triphosphate for Mg^2+^ ions and inhibits transcription and translation by sequestering them. ${{\rm{H}}}_{2}{{\rm{PO}}}_{4}^{-}$ was removed notably faster from the chamber under isothermal conditions compared with sfGFP owing to the lower diffusion coefficient of the latter. Furthermore, if a thermal gradient was applied, sfGFP remained trapped and fast-diffusing phosphate was removed with a delayed onset (Fig. 2b).

Next, we investigated whether the TX-TL reaction could be simultaneously activated and confined (Fig. 2c). For this purpose, the chamber was prefilled with a TX-TL solution without amino acids and tRNAs, and thus unable to synthesize proteins. To probe for protein synthesis, amino acids and tRNAs were continuously supplied from the top of the chamber at a rate of 11 µl h^–1^ for 22 h. Under isothermal conditions (37 °C), the sfGFP concentration quickly rose at the top where the feeding solution entered the chamber, reaching a maximum of 0.53 µM, whereas no expression was observed at the bottom of the chamber due to limited diffusion of the feedstock molecules. However, this left the product at the upper chamber highly susceptible to the outward-directed flow, leading to a steady drop in the synthesized sfGFP concentration. By contrast, applying heat flows under otherwise identical conditions resulted in a strong sfGFP fluorescence signal at the bottom of the chamber, indicating its expression protected from the harmful flow-through at the top. The steadily growing sfGFP concentration reached a value of approximately 0.18 µM by the end of the experiment. Therefore, we concluded that a temperature difference can robustly confine sfGFP for extended periods of time. As a result, the upper part of the chamber acts in a similar way as a diluted extracellular fluid, whereas the lower part resembles a crowded cytosol-like compartment capable of using feedstock molecules from the environment.

Using the model developed with retention, a possible feeding mechanism was simulated. ATP was introduced into the chamber at a concentration of 1 mM carried by the same fluid flow as in previous simulations. In the chamber, ATP was continuously accumulated and converted to ${{\rm{H}}}_{2}{{\rm{PO}}}_{4}^{-}$ according to a Michaelis–Menten kinetic equation^45^ simulating, for example, a transcription reaction. The results of the simulation showed that the ${{\rm{H}}}_{2}{{\rm{PO}}}_{4}^{-}$ present in the chamber before the start of the fluid flow is gradually removed from the trap, after the flow has been started, much faster than the background production (Fig. 2d, blue line). After approximately 800 h, the ${{\rm{H}}}_{2}{{\rm{PO}}}_{4}^{-}$ concentration reaches a steady state in which it is produced and removed from the chamber at similar rates. By contrast, ATP is introduced and retained in the thermal trap, leading to a steady increase in concentration even as it is continuously consumed by the reaction (Fig. 2d, brown line).

### Thermally induced cellular activity

Living cells, as well as in vitro TX-TL systems, require optimized concentrations of their molecular constituents to operate efficiently^14,15^. Cells actively maintain the working concentration of their components by means of chemical energy (ATP) and membrane transport proteins. We next tested whether a similar process could be solely driven by a temperature difference. To that end, we assessed (1) whether all the components of a TX-TL system could be co-accumulated by a temperature difference and (2) whether the accumulation would be strong enough to activate transcription and translation reactions starting from a dilute, inactive TX-TL system. Normally, the TX-TL reaction has an inherent robustness against slight changes in the concentrations of its components. However, drastic changes in composition usually lead to the inactivation of the reaction^46,47^.

The accumulation experiment shown in Fig. 2a gave us an initial indication of the relative and absolute accumulation of sfGFP in our experimental setup. We found a 25-fold relative accumulation, calculated as the ratio of sfGFP fluorescence between the bottom and top of the chamber (BOT/TOP). This value specified the magnitude of the concentration imbalance created by the temperature difference along the height (longitudinal axis) of the chamber. By contrast, we detected a 3-fold absolute accumulation of sfGFP at the bottom of the chamber. This value corresponds to the ratio of sfGFP fluorescence between the end time point and the start of the experiment (BOT_final/BOT_start) and indicates the enrichment of protein at the bottom of the chambers with respect to the initial state.

On the basis of these results, we wondered whether all the other components of a TX-TL system could be similarly accumulated under the same conditions. To address this, we probed the accumulation of ions, amino acids, ribonucleotides, DNA and proteins by measuring their relative and absolute accumulations, starting from one-third of their working concentration inside a closed chamber.

**Fig. 3:**
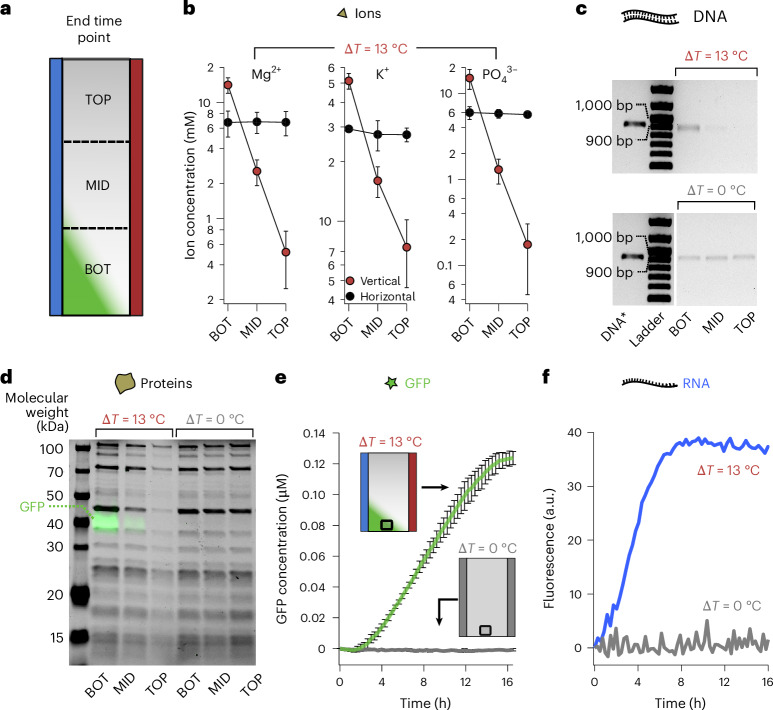
Temperature difference triggers cellular activity. **a**, Schematic of a thermophoretic chamber subjected to a temperature difference (27–40 °C). After 16 h of incubation, the top (TOP), middle (MID) and bottom (BOT) fractions were freeze extracted and sampled for analysis. Chamber controls devoid of accumulation were incubated isothermally (Δ*T* = 0 °C) to switch off thermophoresis or rotated 90° into a horizontal orientation with (Δ*T* = 13 °C) to stop thermal convection. **b**, Ion concentration was probed by chromatography. Mg^2+^, K^+^ and ${{\rm{PO}}}_{4}^{3-}$ showed a strong thermophoretic response with 30-, 7- and 85-fold relative accumulation and 2.5-, 2- and 2.7-fold absolute up-concentration at the bottom of the chamber. The data are represented as the mean value of triplicate experiments ± standard deviation. **c**, Agarose gel shows a 2.7-fold absolute accumulation of the linear DNA template encoding sfGFP used for the experiments. DNA* is the linear DNA fragment as a migration reference. **d**, Merged image of a sodium dodecyl sulfate polyacrylamide gel. The black bands are PURExpress proteins visualized with stain-free technology (Bio-Rad). The relative and absolute accumulations of the total protein were, on average, 6.4- and 1.8-fold, respectively. The green bands correspond to the intrinsic fluorescence of sfGFP. **e**, sfGFP fluorescence kinetics measured at the bottom of the chambers (black squares) from 3-fold-diluted TX-TL reactions incubated with and without a temperature difference (green and grey, respectively). Gene expression was only observed when the temperature difference was applied. The data are represented as the mean value of triplicate experiments ± standard deviation. **f**, Transcription from DNA to RNA was separately visualized using a PCR fragment coding for F30 Broccoli aptamer, showing RNA concentration by fluorescence. The observed accumulation rose faster, probably due to faster transcription, but also from a superior thermal accumulation of RNA at the bottom of the chamber. No signal could be detected in isothermal conditions, suggesting no active transcription.

After 16 h of incubation, we froze the chamber content and sectioned the frozen samples into three equal parts (top, middle and bottom), as detailed and tested in a previous study^40^. Then, we analysed each section by gel electrophoresis and chromatography (Fig. 3a). Bands from agarose and denaturing sodium dodecyl sulfate polyacrylamide gels corresponded to the average amount of DNA and protein from each of the three sections of the chamber.

Ions involved in transcription and translation^48,49^ showed a strong thermophoretic response. Relative accumulations were 30-fold for Mg^2+^, 7-fold for K^+^ and 70-fold for ${{\rm{PO}}}_{4}^{3-}$ (Fig. 3b). The observed variation in relative accumulation was due to the charge and size dependencies of ionic thermophoresis^50^. At the bottom of the chambers, 2.5-, 2- and 2.7-fold absolute accumulations were obtained, respectively, for the same ions, averaged over the extracted bottom section (Supplementary Fig. 9a).

Comparable absolute fold accumulations were obtained for amino acids and ribonucleotides (Supplementary Table 1) and for DNA, where a 2.7-fold absolute accumulation was obtained for the bottom fraction (Supplementary Fig. 9b). The relative accumulation of DNA was estimated to be larger than 40-fold as the depletion at the top was close to the background DNA signal levels (Fig. 3c).

The protein components of the TX-TL system, which considerably differ in molecular weight, showed coherent accumulation patterns as measured by single-band quantification from polyacrylamide gels with 2.4- to 23-fold relative accumulations resulting in an average value of 8.6-fold (Fig. 3d and Supplementary Fig. 9c). The absolute accumulation at the bottom fraction ranged from 1.3-fold to 2.3-fold with an average value of 1.9-fold (Supplementary Fig. 9c). In general, the thermophoresis of proteins is known to be comparably small^51^. Moreover, the similar accumulation patterns of most TX-TL proteins might also reflect their co-localization during coupled transcription and translation reactions including more than 50 ribosomal proteins that are integral parts of the 50S and 30S ribosomal subunits.

Overall, the concentration of ions, amino acids, ribonucleotides, DNA and proteins followed an exponential distribution along the chamber height. Therefore, the local concentration at the very bottom of the chamber was notably higher than that observed experimentally, which corresponded to the average value obtained over 33% of the chamber volume. As expected, non-accumulating isothermal or horizontally oriented control chambers did not show an effective accumulation of TX-TL components (Fig. 3b,d and Supplementary Fig. 9).

Finally, we assessed whether similar levels of ion, amino acid, ribonucleotide, DNA and protein accumulations could lead to the synergistic transcription–translation activation and, therefore, sfGFP synthesis. We started from a TX-TL solution with a 3-fold lower concentration of its components. At such a reduced concentration, no detectable sfGFP expression was found when incubated isothermally at 37 °C (Fig. 3e (grey), Supplementary Fig. 10a and Supplementary Video 2), in agreement with a previous report^14^. The fluorescent protein was neither detected at 27 °C nor 40 °C (Supplementary Fig. 11). Intriguingly, the application of a temperature gradient led to a strong expression of sfGFP at the bottom of the chambers (Fig. 3e (green) and Supplementary Video 1) by the combined co-accumulation of TX-TL components. Furthermore, as expected with the observed accumulation of ribonucleotides (Supplementary Table 1), the recovery of transcription from a similarly diluted TX-TL reaction was confirmed separately with the Broccoli aptamer^52^ (Fig. 3f, green).

The thermogravitational accumulation dependence on chamber thickness was experimentally confirmed by measuring the protein accumulation and sfGFP synthesis along the height of the thermophoretic chambers with different thicknesses (Supplementary Fig. 4b,c). As predicted by the model, we found only minor synthesis of sfGFP for a thinner 125-µm chamber, whereas at 250 µm, no accumulation of proteins and, therefore, no sfGFP synthesis could be observed.

Time-resolved gel and chromatography analyses revealed that DNA and protein accumulations preceded the onset of sfGFP synthesis for several hours, suggesting that the accumulation kinetics of small-molecular-weight components such as ions and amino acids are rate limiting for the initiation of protein synthesis (Supplementary Fig. 12).

In addition, to test the limits of what can be assembled/accumulated with thermogravitational accumulation, we attempted to recover the synthesis of sfGFP from a more complex molecular mixture such as the one provided by a cell-free extract (Supplementary Fig. 13). In this case, the proteins did not accumulate substantially at the bottom fraction of the chamber, possibly due to a higher viscosity of the cell-free extract. sfGFP, however, was present more abundantly at the bottom, decreasing in intensity non-exponentially to the top of the chamber, in contrast to model expectations. This suggests that the reaction was active, but the accumulation mechanisms were markedly more complex. To further clarify the relationship between the accumulation of TX-TL components and sfGFP synthesis, 3-fold-diluted reactions were mixed with glycerol to a final concentration of 10% or 20% (v/v). The solutions were incubated under the same conditions as in the previous experiments. In both cases, the onset of sfGFP synthesis was delayed compared with the control experiment (Supplementary Fig. 16), probably due to slower convection currents. Interestingly, higher levels of sfGFP fluorescence were detected at the bottom of the chamber for 20% glycerol compared with 10%, but both were below the levels without added glycerol. Since the solutions did not reach the plateau phase during the 18-h incubation period, it is possible that the overall sfGFP yield is higher than in standard reactions.

Furthermore, to better understand the kinetics of the system, additional experiments were performed in which, after an initial 18-h incubation period in a thermal gradient and sfGFP synthesis, the gradient was turned off and the chamber was isothermally incubated at either 27 °C or 40 °C. The decrease in concentration at the bottom of the thermal trap due to sfGFP diffusion was observed for another 6 h. As expected, diffusion was much faster at 40 °C than at 27 °C, as indicated by the rate at which the fluorescence intensity decreased compared with the beginning of the isothermal incubation period (Supplementary Fig. 17). Additionally, we simulated the isothermal conditions at 27 °C for a longer period (10,000 h) and found that a uniform distribution of sfGFP is reached after approximately 3,000 h. This highlights the longevity of accumulation by thermophoresis.

Interestingly, it was not necessary to tune the individual initial TX-TL component concentrations to obtain an operational TX-TL system since the strength of thermophoretic accumulation is known to be independent of the starting molecular concentrations^33,34,50,53^ and led to similar absolute fold accumulations. The coordinated accumulation achieved a sufficiently high concentration of all the molecular components to activate protein synthesis. Protein concentration calibrations in custom-made PURE showed that the overaccumulation of individual proteins still resulted in a functional TX-TL reaction, but without increasing the protein yield^54^, demonstrating a robustness that probably helped our experiments. Within our experimental setting, the recovery of sfGFP was observed up to a 4-fold-diluted TX-TL reaction. Higher dilutions led to no measurable signal (Supplementary Fig. 10b).

### Thermal protocells potential and implications

Overall, our results showed that the heat-flow-driven accumulation of ions, amino acids, ribonucleotides, DNA and proteins led to a confined functional gene expression system at the bottom of the compartment, insulated by the absolute thermophoretic accumulation from the top of the chamber. Consequently, the prebiotic period in which this accumulation mechanism would be the most relevant would be from the formation of the first biomolecules to the encapsulation of these biomolecules in lipid vesicles. At this stage, dilution should not be as much of a problem for the nascent prebiotic networks. Furthermore, thermogravitational accumulation may even aid encapsulation by accumulating lipid precursors above the critical vesicle concentration^36^. In our fluorescence images, we could not find evidence for coacervate formation or a liquid–liquid phase separation^16^. The molecules are not accumulated to the extent that a phase transition is triggered, and we rather observe cooperative concentration enhancement in the PURE expression system by the thermophoretic forces.

In future experiments, higher thermophoretic chambers are likely to overcome higher dilutions of the TX-TL reaction. However, one limitation not related to the accumulation strength of the thermogravitational mechanism is the dissociation of ribosomes at high dilutions. The *E. coli* ribosomes used in the TX-TL reaction are highly dependent on the Mg^2+^ concentration. The more the reaction is diluted, the greater the disassembly of the ribosomal subunits, to a point where reversibility by accumulation is also limited^55,56^. It should also be noted that the absolute accumulation of molecules was limited in a closed chamber. Further geometrical optimizations would need to be undertaken to confine and maintain the activity of the TX-TL reaction for increasingly longer periods of time by supplying nutrients to the reaction and removing waste molecules. Such an open system with an external supply of TX-TL components and continuous accumulation could achieve a substantially higher absolute concentration, potentially reaching values known from biological cells^57^. Additionally, the temperature difference offers dynamic and direct access to the inner concentrations of the components of the thermally assembled protocell, enabling multiple modes for molecular exchange including feedback, signalling, feeding and chemical communication.

## Conclusion

Living cells are highly organized, out-of-equilibrium chemical systems that sustain themselves by consuming fuel sources through complex metabolic processes. Attempts to construct such systems have mainly relied on the insertion of molecular components into lipid vesicles. However, little progress has been made in harnessing fuel sources to maintain the low-entropy state that characterizes biology. Rather than focusing on overcoming the difficulties of reconstituting functional membrane proteins and linking their activity to the maintenance of the cell, we used the energy of thermal gradients to self-organize a complex mixture of molecules into a membraneless, cell-like structure.

We found that the optimal thickness of the rock pore for accumulation is between 100 µm and 200 µm for a vertical thermal trap. This optimal thickness is larger, reaching millimetres for inclined traps^58^. The optimal trap is independent of chamber height. Sections of non-optimal thickness are bridged by diffusion and can be compensated by a higher chamber^37^. Given the variety of pore shapes and sizes found in volcanic rocks and considering our results and those of previous studies, thermogravitational accumulation may prove to be a very promising candidate for large-scale accumulation on a prebiotic Earth^35^.

It was unclear that the wide range of molecules required for a biological cell could be solely accumulated by a temperature difference, operating under physiologically high salt concentrations and near-crowding conditions, as well as reaching the concentrations needed to sustain transcription and translation. However, our results now show that it is feasible and offers many possibilities to create a network of interconnected thermal cells using flow or diffusion^26^, moving closer to the ultimate goal of controlling biological evolution in networks of protocells.

## Methods

### Static accumulation experiments

The TX-TL system used in the experiments was the commercially available PURExpress in vitro protein synthesis kit (E6800, NEB), which was prepared following the manufacturer’s instructions. Standard 1× concentrated reactions contained for 30 µl:100 ng (5.9 nM) of linear polymerase chain reaction (PCR) fragments coding for sfGFP (or F30 Broccoli aptamer), RNAse inhibitor (20 units), 10 µl of solution A, 7.5 µl of solution B and MilliQ water. For transcription experiments, 4.5 µl of 200-µM DHFBI-1T was also added (final concentration, 30 µM). Note that these reactions were assembled with an excess of 20% water, which leads to no notable decrease in the in vitro protein synthesis according to the PURExpress manual. The DNA templates used for the reactions were amplified using the Q5 Hot Start High-Fidelity 2× master mix (M0494S, NEB) from plasmids, encoding for either sfGFP or F30 Broccoli, provided by Laura I. Weise (Supplementary Table 2). Inactivation of the reaction mixture was ensured by mixing 30 µl of TX-TL solution with 60 µl of MilliQ water.

The thermal trap was assembled according to the scheme shown in Supplementary Fig. 1 using custom-made components. First, a 25-µm graphite foil (Panasonic) was laid on an aluminium base (Star Rapid) followed by a 0.5-mm-thick sapphire plate (KYBURZ). The reaction chamber was cut from a 170-µm fluorinated ethylene propylene (FEP) foil (Holscot) using a plotter (CE6000-40 Plus, Graphtec) and added to the stack. Next, a second sapphire plate with a thickness of 2 mm was laid over the FEP foil before the entire stack was topped with a steel frame, fixed in place using six steel screws at a torque of 0.26 N m. The sapphires were previously coated on both sides with PROSURF MT-5 (Surfactis). The excess surfactant was removed using cotton swabs (414004-518, VWR). The assembly of the thermal trap was conducted in a laminar-flow chamber to minimize dust incorporation.

The chamber was first filled with 3M Novec 7500 Engineered Fluid from a glass syringe through FEP tubes with an inner diameter of 0.25 µm, connected to the trap using plastic screws. The oil syringe was removed and 60 µl of TX-TL solution was carefully filled from the opposing side to avoid the formation of air bubbles. After the tubes were sealed with plastic caps, the heater was mounted on the front side of the trap assembly, separated from the sapphire plate by another graphite foil (200 µm). The heater was fixed using four steel screws at a torque of 0.16 N m. Next, the trap was mounted on an aluminium block using two FEP screws at a torque of 0.4 N m. Another graphite foil ensured an even contact between the trap and the aluminium block, which was cooled to 10 °C using a water bath. The temperature at the front of the trap was maintained at 54 °C through the heater. The reactions were incubated up to 24 h depending on the experiment. For the control experiments, the traps were either flipped 90° to a horizontal orientation (eliminating convection) or incubated isothermally at 37 °C using modified heaters that were in contact with the back aluminium base (providing even heat distribution). Image acquisition, temperature control and image analysis for both sfGFP and F30 Broccoli experiments were performed using custom-made LabView 2014 programs, described in detail in the ‘Material and methods’ section in Supplementary Information.

### Flow experiments

For the flow retention and activation experiments, the microfluidic geometry was slightly modified to allow the inflow and outflow of water or feeding solution (amino acids and tRNAs provided in the kit; E6840S, NEB) at the top of the thermophoretic chambers through a 1-mm-diameter open channel connecting the inlet and outlet insertion holes. The schematic of the microfluidic geometry is shown in Supplementary Fig. 7. Sample loading with either full or amino-acid- and tRNA-deficient TX-TL reaction (PURExpress Delta aa, tRNA kit, E6840S, NEB) was conducted directly at the setup with low-pressure syringe pumps (neMESYS 290N, CETONI) mounted on a low-pressure 29:1 module (NEM-B101-03 A, CETONI).

### Freeze extraction

After incubation, the traps were removed from the experimental setup and cooled in a −80 °C freezer for at least 2 h. The now frozen reactions were kept cold in a Styrofoam box on an aluminium block that was also precooled to −80 °C. Dry ice was also added to reduce the condensation of water on the sapphire plates. The traps were opened and one of the sapphires was carefully removed to leave the frozen reaction volume intact. The reaction chamber was split into three equal parts and selectively molten by gradually pushing the sapphire over a hot aluminium block. The fractions were then simultaneously extracted as they melted using a pipette and transferred to the reaction tubes.

### Analysis of trap fractions

Trap fractions were subsequently subjected to DNA, protein and ion composition analyses. To visualize the accumulation of proteins, 2 µl of the sample was mixed with 3 µl of ROTI-Load 1 (4× concentrated, reducing; K929.1, Roth) and to 12 µl with MilliQ water. The samples were subsequently incubated at 65 °C for 2.5 min. Then, 10 µl of each fraction was filled in the pockets of an AnyKD Mini-PROTEAN TGX stain-free precast gel (4568126, Bio-Rad) together with 1.5 µl of unstained protein ladder (26630, Thermo Scientific). The gel was run at 200 V in a 1× Tris–glycine–sodium dodecyl sulfate buffer for approximately 40 min. Gel images were acquired in a ChemiDoc MP Imaging System (Bio-Rad) using the provided stain-free protein gel and Alexa Fluor 488 protocols with the rapid auto-exposure option. Additionally, gels were stained using 50 ml of 0.6× SYPRO Ruby (S4942, Sigma) overnight under constant rocking and imaged.

The DNA extraction from the freeze-extracted samples was conducted as follows. First, to digest all the RNA species present in the TX-TL solutions, 5 µl of the freeze-extracted samples were combined with 10 units of RNAse 1 (EN0601-Thermo Fisher Scientific) and 2 µg per 50 units of an RNAse A/T1 mix (EN0551-Thermo Fisher Scientific). The solution was incubated for 30 min at 37 °C. Subsequently, proteins were digested with eight units of Proteinase K (P8107S) for 1 h at 37 °C. Both incubations were conducted in a ProFlex PCR system (4484073, Thermo Scientific). DNA purification was done with the Monarch PCR and DNA clean up kit (T1030S, NEB) following the manufacturer’s instructions for dsDNA fragments smaller than 2 kb. Samples were eluted in 6 µl of TE elution buffer (10 mM Tris, 0.1 mM EDTA, pH 8.5). Then, 5 µl of DNA samples was mixed with 1 µl of TriTrack 6× loading dye (R1161, Thermo Scientific) run on a 1% TBE Wide Mini ReadyAgarose precast gel (1613028, Bio-Rad) prestained with ethidium bromide together with a GeneRuler 100-bp plus DNA ladder (SM0323, Thermo Scientific) at 150 V for approximately 30 min in TBE buffer. Last, the gels were imaged using the ethidium bromide setting and analysed using ImageLab 6.1 (Bio-Rad), as specified in Supplementary Information.

For the analysis of ions in the reaction fractions, 1.5 µl from each freeze-extracted sample was diluted in 520 µl of water (00612, Supelco). Samples were injected using an autosampler (AS-DV, Thermo Fisher Scientific) into both a cation (Dionex Aquion, Thermo Fisher Scientific) and an anion ion chromatography system (Dionex Integrion, Thermo Fisher Scientific). The cationic system consisted of an analytical column (Dionex IonPac CS12A), guard column (Dionex IonPac CG12A) and suppressor (Dionex CDRS 600). The following method was used to separate Mg^2+^ and K^+^ ions: flow of 0.15 ml min^–1^, isocratic elution with 7.5 mM of methanesulphonic acid, 5 mA of suppression, cell temperature of 45 °C and column temperature of 50 °C. The anionic system comprised an analytical column (Dionex IonPac AS16 2 mm), guard column (Dionex IonPac AG16 2 mm), suppressor (Dionex ADRS 600 2 mm), eluent generator (EGC 500 KOH) and trap column (Dionex CR-ATC 600). The method used for anion separation was as follows: flow of 0.30 ml min^–1^, isocratic elution with 27.5 mM of KOH, 21 mA of suppression, cell temperature of 45 °C and column temperature of 50 °C. Eluted ions and anions were measured with a conductivity detector (DS6 Heated Conductivity Cell). The data were analysed using Chromeleon 7.2.10 (Thermo Fisher Scientific). Peak analysis was done by automatic peak integration and manually adjusted when needed. Standard concentration curves were measured using the Dionex Combined Six Cation Standard-I (040187, Thermo Fisher) and Dionex Combined Seven Anion Standard II (057590, Thermo Fisher).

The materials and methods are described in more detail in Supplementary Information, including the preparation and description of the DNA construct used in this study, the in vitro transcription–translation reactions, the description of the thermophoretic chambers and setup used for the static and flow experiments, and the freeze-extraction protocol used to extract samples from the thermophoretic chambers for further analysis. Further information is given on the DNA extraction protocol and gel electrophoresis, protein gel electrophoresis and analysis, ion chromatography to quantify the concentration of ions, amino acid and nucleotide detection, fluorescence analysis, fold accumulation calculations and the finite element model of the thermogravitational accumulation of sfGFP.

### Reporting summary

Further information on research design is available in the Nature Portfolio Reporting Summary linked to this article.

## Online content

Any methods, additional references, Nature Portfolio reporting summaries, source data, extended data, supplementary information, acknowledgements, peer review information; details of author contributions and competing interests; and statements of data and code availability are available at 10.1038/s41567-025-02935-4.

## Supplementary information

Supplementary Information Supplementary Figs. 1–17 and Tables 1 and 2.

Reporting Summary

Supplementary Video 1 sfGFP expression of a diluted PURE reaction under a temperature gradient of 27–40 °C for 16 h.

Supplementary Video 2 A diluted PURE reaction incubated isothermally at 37 °C for 16 h.

## Data Availability

All data are provided with this manuscript and its Supplementary Information. Source data are available via the Open Data LMU repository at 10.5282/ubm/data.606. More information can be requested by contacting the coresponding author D.B. via the email dieter.braun@lmu.de.
