# Supplementary material for: Membraneless protocell confined by a heat flow
Source: Nat Phys. 2025 Jun 26;21(8):1303–10. doi: 10.1038/s41567-025-02935-4 (PMC12343290; doi:10.1038/s41567-025-02935-4)
Supplement: Supplementary file 1 — Supplementary Figs. 1–17 and Tables 1 and 2. [file 41567_2025_2935_MOESM1_ESM.pdf]

nature physics

Supplementary information https://doi.org/10.1038/s41567-025-02935-4

Membraneless protocell confined by a heat flow

In the format provided by the authors and unedited

Contents

Material and Methods 3
Preparation and description of the DNA construct used in this study 3
In vitro transcription-translation reactions 3
Thermophoretic chambers and setup...4
Flow experiments 5
Freeze-extraction protocol 5
DNA extraction and gel electrophoresis...6
Protein gel electrophoresis and analysis...6
Ion chromatography 7
Amino acid detection 7
Nucleotide detection 7
Fluorescence analysis..8
Fold accumulation calculations 8
Finite element model of the thermogravitational accumulation of sfGFP 8
(a) Determination of the thermophoretic properties of sfGFP:..9
(b) Determination of the optimal thickness of the thermophoretic chambers: 10
(c) Retention of sfGFP against a water flow by thermograviational accumulation: 10
(d) Phosphate waste removal under a water flow regime 11
(e) ATP accumulation and conversion to phosphate 11
Fig. S1. General overview of the experimental setup...12
Fig. S2. Gene expression test in PROSURF coated versus uncoated thermophoretic chambers. . 13
Fig. S3. sfGFP in vitro protein synthesis works inside thermophoretic chambers incubated isothermally. 14
Fig. S4. Assessing the optimal thickness of the thermophoretic chamber using finite element simulations...15
Fig. S5. Temperature differences across thermophoretic chambers do not impair in vitro gene expression and accumulate sfGFP inside thermophoretic chambers. 16
Fig. S6. Temperature dependent sfGFP expression kinetics in test tubes.................................17
Fig. S7. Temperature differences across thermophoretic chambers retain and protect sfGFP against dilution...18
Fig. S8. Estimation of model parameters for sfGFP retention against a flow of water. 19
Fig. S9. Accumulation of ions, DNA and proteins and determination of absolute accumulation ratios. 21
Fig. S10. Assessing in vitro sfGFP expression from diluted TX-TL reactions inside thermophoretic chambers incubated under isothermal conditions. 22

Fig. S11. sfGFP expression recovery from inactive 0.33x concentrated TX-TL reactions.............23
Fig. S12. Accumulation kinetics of DNA, proteins, ions and amino acids. 24
Fig. S13. Synthesis and accumulation of sfGFP from diluted cell lysate in a thermophoretic chamber..25
Fig. S14. Calibration curves used for converting fluorescence units into sfGFP concentrations...26
Fig. S15. Accumulation of different concentrations of purified sfGFP 27
Fig. S16. Synthesis and accumulation of sfGFP in PURE reactions with increased viscosities. 28
Fig. S17. Diffusion of sfGFP after synthesis at different isothermal temperatures......................29
Table S1. Thermogravitational accumulation of amino acids and ribonucleotides. 30
Table S2. DNA plasmid and primer sequences. 32
Movie S1. sfGFP expression recovery inside a thermophoretic chamber subjected to a temperature difference..32
Movie S2. Unfunctional TX-TL reaction inside a thermophoretic chamber isothermally incubated at 37 °C. 32
SI References...33

Material and Methods

Preparation and description of the DNA construct used in this study

Linear DNA fragments coding for superfolder green fluorescent protein (sfGFP) and F30 Broccoli aptamer were obtained by PCR amplification from plasmids pGEMT-MS2-sfGFP and pUCIDT_MS2_F30Bro respectively (Sequences shown in Supplementary table S2) which were previously used in[1]. Primers used for the PCR reactions are shown in Supplementary table S2. PCR reactions were assembled with 1 ng of plasmid template, 1x final concentration of Q5® Hot Start High-Fidelity 2x master mix, 0.5 µM final concentration of both forward and reverse primers and Milli-Q water up to 100 µL. The thermocycling protocol was performed in a ProFlex™ PCR-system (4484073, Thermo Scientific) and consisted of an initial denaturation step of 30 s at 98 °C, followed by 35 cycles of (denaturation: 98 °C-30 s/ Annealing: 65 °C-20 s / Extension: 72°C-20 s) and a final extension step at 72 °C for 2 min.

The length of the PCR fragments was analyzed by gel electrophoresis on 1 % w/v agarose (A96539, Sigma) gel stained with 1x SYBR safe (S33102, Invitrogen) and run with 1x TBE buffer. A GeneRuler 100 bp plus DNA ladder (SM0323, Thermo Scientific) was used as a reference. Subsequently, to digest the plasmid template, the PCR reactions were incubated with 40 units of DpnI (R0176S-NEB) in 1x CUTsmart® buffer at 37 °C for 45 min and 80 °C for 20 min to inactivate the enzyme. The PCR reactions were then purified using the Monarch® PCR and DNA clean up kit (T1030S, NEB) following the manufacturer protocol for dsDNA fragments smaller than 2 kb and eluted in 12 µL of TE elution buffer (10 mM Tris, 0.1 mM EDTA, pH 8.5). The DNA concentration was quantified with a NanoDrop ONE^{C} (Thermo Scientific) and the purified linear PCR fragments were stored at – 20 °C until further use.

In vitro transcription-translation reactions

In vitro transcription-translation reactions were conducted with PURExpress in vitro protein synthesis kit (E6800, NEB). Solution A and solution B were aliquoted in 0.2 mL sample tubes and stored at -80 °C before use. Reactions were assembled on ice following the manual instructions of the provider. In short, standard 1x concentrated reactions contained for 30 µL: 100 ng (5.9 nM) of linear PCR fragments coding for sfGFP (F30 Broccoli aptamer), RNAse inhibitor (20 units), 10 µL of solution A, 7.5 µL of solution B and MilliQ-water. For transcription experiments 4.5 µL 200 µM DHFBI-1T were also added (30 µM final concentration). Note that these reactions were assembled with an excess of 20% water, which leads to no significant decrease in in vitro protein synthesis according to the PURExpress manual. TX-TL dilutions were prepared by further dilution of the 1x stock with Milli-Q water.

For the viscosity experiments, a 50% glycerol solution (Merck) was added in different amounts instead of water thereby bringing the final concentration of glycerol to 20% or 10%.

Purified sfGFP (provided by the lab of Sheref Mansy) was mixed with a 3-fold diluted PURE reaction to 0.34, 0.1 and 0.034 µM final concentration. In these experiments DNA was left out in order to not induce any additional sfGFP production by the PURE itself.

Cell-free extract experiments were conducted using NEBExpress® Cell-free *E. coli* protein synthesis system (E5360, NEB). The reactions were assembled on ice by mixing: 14.4 µL S30 Extract, 30 µL Protein Synthesi Buffer (2x), 1.2 µL T7 Polymerase, 1.2 µL RNAse inhibitor Murine, which were supplied with the kit and 250 ng linear PCR fragment. Furthermore 1.2 µL NEBExpress® GamS Nuclease Inhibitors (P0774S, NEB) were also added to stabilize the linear DNA fragment. Finally, the reaction was filled to 180 µL with MiliQ-water resulting in a 3-fold diluted solution.

Thermophoretic chambers and setup

Component parts and assembly of thermophoretic chambers. The custom-made thermophoretic chambers components are illustrated in (Fig. S1A). All parts were designed with Inventor Professional 2021 (Autodesk). Aluminum and steel components were manufactured by (Star Rapid). Details on thermophoretic chamber design and development are given in[2]. The microfluidic geometries used in the experiments are shown in (Fig. S1B and S7) and were cut into 170 µm thick FEP-foil (Holscot, Netherlands) with an industrial cutter plotter (CE6000-40 Plus, Graphtec, Germany).

Thermophoretic chambers were assembled inside a laminar flow cabinet (AURA mini, EuroClone) to avoid potential dust particles deposition in any of the surfaces. The assembly was conducted with the following steps. First, a heat conducting foil (EYGS091203DP, graphite, 25µm, 1600 W/mK, Panasonic, Japan) was placed on top of an aluminum base. Then the FEP foil was sandwiched between two sapphire plates (Kyburz, Switzerland). Before the assembly, the sapphire plates were coated with PROSURF MT-5 (Surfactis) which is a perfluoropolyether repellent compound dissolved in a fluorinated solvent. ~ 200 µL of PROSURF MT-5 were applied on top of each surface, dried at ambient temperature and homogeneously distributed with a cleanroom knit wiper (414004-518, VWR). The coating allowed the recovery of gene expression from 0.33x concentrated TX-TL reactions, as opposed to untreated sapphires (Fig. S2). This indicated the presence of unwanted interactions between the untreated sapphire surfaces and the TX-TL components e.g. proteins. The sapphire-FEP-sapphire parts were held together and fixed to the aluminum base with a steel frame support with 6 steel screws. A 0.26 (Nm) torque was applied in each screw to ensure homogenous force distribution. Then the thickness i.e. the distance across the two sapphire plates was measured with a confocal micrometer (CL-3000 series with CL-P015, Keyence, Japan) at five different positions. Thicknesses of 170 ±5 µm were consistently measured for all the assembled chambers with a maximum of 5 µm difference along the height (longitudinal axis, 35 mm) of a single chamber.

Sample loading. 4 laser-cut holes (1 mm Ø) on the back (cold) sapphire allowed for loading access. The following materials used for loading were purchased from Techlab. Two FEP tubings (0.25 um inner Ø, KAP 100.966) each one coupled with one plastic screw (VBM 100.826, VBM 100.828) and one ferrule (VBM 100.632) at each end were inserted through the aluminum base access holes and directly connected to the surface of the back sapphire at the inlet and outlet insertion holes. Then thermophoretic chambers were pre-filled with fluorinated oil (3M™ Novec™ 7500 Engineered Fluid) to check for tightness and remove any residual air inclusion that would disturb thermogravitational accumulation. Prior sample loading, chambers were incubated for ~ 30 min at 4 °C. This pre-incubation step was necessary to pre-cool the chambers to avoid the onset of gene expression, from 1x concentrated TX-TL reactions, during the loading process. A double connector (UP P-702-01) was used as bridge connection between [1] a 250 µL syringe (2606814, Göchler-HPCL syringes) connected to another FEP tubing (1 mm inner Ø, KAP 100.969) manually pre-filled with fluorinated oil and 50 µL of TX-TL solutions and [2] the FEP tubing connected to the inlet insertion hole. Such connection ensured gas-free conditions while loading the chambers. The sample was carefully loaded through the inlet while the outlet was left open, until the central column of the geometry was completely filled. Finally the tubing end parts were tightly closed with end caps (Tefzel cap for 1/4-28 Nut, UP P-755) to avoid evaporation of the TX-TL solution during the duration of the experiments.After loading, another heat conducting foil (EYGS0811ZLGH, graphite, 200µm, 400 W/mK, Panasonic, Japan) was placed on top of the front (warm) sapphire and an aluminum support for the heater rods was fixed to the steel frame with a 0.16 (Nm) torque applied to 4 steel screws. Such conformation allowed the direct contact of the heater support with the front (warm) sapphire through the heat conducting foil. The heater support had a rectangular milled slit (3 x 46 mm) allowing optical access to the chamber by fluorescence microscopy.

Setup. The custom-built setup was composed of an aluminum multi-support for multiple thermophoretic chambers, a custom-made microscope and a custom-made program (LabVIEW version, 2014). Microscope parts are described in detail in the caption of Figure S1. The microscope

was mounted on top of an aluminum stage coupled with three microstep motors that allowed the movement of the microscope in three dimensions. Two motors (NEMA23, Simac Electronics GmbH, Germany) allowed the move in (x,y) and another motor (Thorlabs, Germany) allowed the move in (z). The multi-modular custom-made LabVIEW program allowed the parallel and coordinated control of different functionalities of the setup via user defined interconnected module workflows. For example, the measuring workflow allowed the fluorescence acquisition at specific selected positions of multiple chambers by controlling the step motors via an ardunio board and also controlled the LED illumination parameters and image acquisition. The temperature workflow controlled the temperature of the heater rods via another Arduino board and the fluid-flow workflow allowed the control of the syringe pump's flow rate for the retention experiments. Each module workflow was triggered individually or in a concerted way depending on the experimental needs.

Temperature settings. To apply temperature differences to the thermophoretic chambers a water bath (Grant TXF200-R5, UK) was set to 10 °C and the heaters were set to 54 °C resulting in a temperature difference between ~27 °C - 40 °C. To estimate these values, first the temperature outside the sapphire plates was measured with a thermistor (GTH 170 , Greisinger, Germany) placed in direct contact to the surface of the (cold) sapphire and a heat imaging camera (SeekShotPro, Seek Thermal, Inc., California, US) measuring the temperature of the (warm) front sapphire. The temperatures on the inside surfaces of the sapphires in contact with the TX-TL solutions were calculated with another custom-made LabVIEW program considering the thermal conductivities $k = 0.62\ W/mK$ for water and $k = 35\ W/mK$ for sapphire plates and the thicknesses of 0.5 and 2 mm for cold and hot sapphire plates respectively. For control chambers incubated isothermally, the aluminum heater supports were modified to completely cover the front of the chambers without touching the front sapphire plates but directly in contact with the aluminum base. In this way, the whole chamber could be kept at a homogeneous and constant temperature, avoiding any temperature gradients.

Flow experiments

For the flow retention and activation experiments the microfluidic geometry was slightly modified to allow the inflow and outflow of water or feeding solution (amino acids and tRNAs, provided in the kit, E6840S, NEB) at the top of the thermophoretic chambers through a 1 mm Ø open channel connecting inlet and outlet insertion holes. The schematic of the microfluidic geometry is shown in (Fig. S7). Sample loading with either full or amino acid and tRNA deficient TX-TL reaction (PURExpress Delta aa, tRNA Kit, E6840S, NEB) was conducted directly at the setup with low pressure syringe pumps (neMESYS 290N, Cetoni) mounted on a low pressure module 29:1 (NEM-B101-03 A, Cetoni). The loading workflow was set with the same LabVIEW program that controlled the imaging acquisition, the temperature control of the heaters and the motorized stage of the microscope.

Freeze-extraction protocol

The freeze extraction protocol was conducted as previously reported in (23) with slight modifications. After each experiment, thermophoretic chambers were removed from the setup and immediately stored inside a -80°C freezer for at least 2 h. Then, the chambers were placed on top of an aluminum block pre-cooled at -80 °C to keep the samples frozen. Chambers were then disassembled by first removing the FEP tubings and steel holder. Then the sapphire-FEP-sapphire sandwich was carefully separated from one of the bottom corners with the help of a razor. The frozen TX-TL solutions remained attached to one or both of the sapphire plates. To avoid water condensation on top of the frozen samples during the extraction, dry ice was placed surrounding the cold aluminum block. Samples were sectioned into three parts with the razor. The length of each section was previously measured with a ruler to equally delimit each fraction. To collect each fraction volume, a second aluminum block (~ 50 °C) was placed next to the cold block and separated by an insulator material. The sapphire plates were gently and slowly pushed from the cold to the warm aluminum blocks to allow the gradual melting of the frozen solutions. The melted

TX-TL reactions were immediately collected by pipetting and stored in Eppendorf tubes at – 80 °C before sampling them for subsequent analysis.

DNA extraction and gel electrophoresis

DNA extraction from freeze-extracted samples was conducted as follows. First, in order to digest all RNA species present in the TX-TL solutions, 5 µL of freeze-extracted samples were combined with 10 units of RNAse 1 (EN0601-ThermoFischer Scientific) and 2µg/50 Units of an RNAse A/T1 mix (EN0551-ThermoFischer Scientific). The solution was incubated for 30 min at 37 °C. Subsequently, proteins were digested with 8 Units of Proteinase K (P8107S) for 1 h at 37 °C. Both incubations were conducted in a ProFlex™ PCR-system (4484073, Thermo Scientific). DNA purification was done with the Monarch® PCR and DNA clean up kit (T1030S, NEB) following the manufacturer instructions for dsDNA fragments smaller than 2kb. Samples were eluted in 6 µL of TE elution buffer (10 mM Tris, 0.1 mM EDTA, pH 8.5).

5 µL of samples were combined with 1 µL of TriTrack 6x loading dye (R1161, Thermo Scientific) and 5 µL were loaded into 1% agarose gels pre-stained with 1x SYBR safe. A GeneRuler 100 bp plus DNA ladder (SM0323, Thermo Scientific) was used as a reference. Gels were run in 1x TBE buffer at 150 V. Image acquisition was done with a ChemiDoc MP Imaging System (Bio-Rad) with rapid auto-exposure (excitation: 302 nm/ emission filter: 590/110 nm). The agarose gel shown in Fig. 3C and Fig. S9A was a 1% TBE Wide Mini ReadyAgarose precast gel (1613028, Bio-Rad) pre-stained with ethidium bromide and imaged with rapid auto-exposure and (excitation: 302 nm/ emission filter: 602/50 nm). DNA bands were quantified with ImageLab 6.1 (Bio-Rad). Lanes and bands were detected automatically and manually adjusted when needed. Global Background subtraction was set by adjusting the disk size according to the sample lane with the lowest signal to noise in the gel and the same disk size value was applied for all the lanes of the same gel.

Protein gel electrophoresis and analysis

The distribution profile of proteins along the thermophoretic chambers was assessed by gel electrophoresis. 3 µL of loading dye (Roti®-Load 1 4x concentrated, reducing; K929.1, Roth) were combined with 2 µL of each freeze-extracted sample from 0.33x concentrated TX-TL solutions and with MilliQ-water up to 12 µL. Samples were heated at 65 °C for 2.5 min inside AccuBlock™ Digital Dry Bath (Labnet). Under these conditions, the fluorescence of sfGFP could still be detected in gel. Subsequently, 10 µL/sample were loaded into either 15 well AnyKD™ Mini-PROTEAN® TGX Stain-free™ precast gels (4568126, Bio-Rad) or 18 well AnykD™ Criterion™ TGX Stain-free™ precast gels (5678124, Bio-Rad) depending on the number of samples loaded per gel. For single band analysis, 10 well 7.5% Mini-PROTEAN® TGX Stain-free™ precast gels (4568024, Bio-Rad) were used to increase the separation between single bands (Fig. S9C, right gel). An unstained protein ladder (26630, Thermo Scientific) was used as reference. Gel electrophoresis was conducted in a Mini-PROTEAN tetracell (1658004, Bio-Rad) or midi Criterion™ cell (1656001, Bio-Rad) with 1x Tris-Glycine-SDS buffer (3060.3, Roth) at 200 V. Gel images were acquired with a ChemiDoc MP Imaging System (Bio-Rad) with rapid auto-exposure. For single band analysis, gels were acquired with optimal auto-exposure. To visualize PURExpress proteins and ribosomal proteins with stain-free technology (Bio-Rad), gels were pre-activated for 5 min and images acquired with (excitation: 302 nm/ emission filter: 590/110 nm). To visualize sfGFP fluorescence (excitation: 460-490 nm/ emission filter: 532/28 nm). Subsequently all gels were stained with 50 mL of a 0.6x SYPRO Ruby (S4942, Sigma) solution following the basic protocol with overnight incubation in a shaker (88861022, Fisher Scientific) at 175 rpm. Stained images were acquired with (excitation: 302 nm/emission filter: 590/110 nm). Protein band quantification was performed with imageLab 6.1 (Bio-Rad) on the gels stained with SYPRO Ruby. Lanes and bands were detected automatically and manually adjusted when needed. Global Background subtraction was set by adjusting the disk size according to the sample lane with the lowest signal to noise in the gel and the same disk size value was applied for all the lanes of the same gel. The molecular weight of single bands (shown in Fig.

S9C, right gel) was extrapolated by linear regression from the reference ladder considering reference bands from 250-30 kDa.

Ion chromatography

To measure the ion concentration profile along the thermophoretic chamber sections, freeze-extracted samples, without prior purification, were subjected to ion chromatography. For the analysis, 1.5 µL from each freeze-extracted sample were diluted in 520 µL of water (00612, Supelco). Samples were injected using an autosampler (AS-DV, ThermoFisher Scientific, USA), simultaneously injecting into a cation IC (Dionex Aquion, ThermoFisher Scientific, USA) and an anion IC (Dionex Integrion, ThermoFisher Scientific, USA). The cationic system consisted of an analytical column (Dionex IonPac CS12A), guard column (Dionex IonPac CG12A) and suppressor (Dionex CDRS 600). The following method was used to separate Mg^{2+} and K^{+} ions: 0.15 ml/min flow, isocratic elution with 7.5 mM MSA (Methanesulphonic acid), 5 mA suppression, cell temperature of 45 °C and column temperature of 50 °C. The anionic system comprised an analytical column (Dionex IonPac AS16 2mm), guard column (Dionex IonPac AG16 2mm), suppressor (Dionex ADRS 600 2mm), eluent generator (EGC 500 KOH) and trap column (Dionex CR-ATC 600). The method used anion separation was: 0.30 ml/min flow, isocratic elution with 27.5 mM KOH, 21 mA suppression, cell temperature of 45 °C and column temperature of 50 °C. Eluted ions and anions were measured with a conductivity detector (DS6 Heated Conductivity Cell). Data was analyzed using Chromeleon 7.2.10 (ThermoFisher Scientific, USA). Peak analysis was done by automatic peak integration and manually adjusted when needed. Standard concentration curves were measured using the "Dionex™ Combined Six Cation Standard-I" (040187 ThermoFisher, USA) and "Dionex™ Combined Seven Anion Standard II" (057590 ThermoFisher, USA).

Amino acid detection

Amino acids were detected by high performance liquid chromatography (HPLC) without prior amino acid purification from each freeze-extracted sample. The HPLC system (Vanquish Core (VC-S01-A-02), ThermoFisher, USA) was equipped with a quaternary pump and a fluorescence detector. To separate the amino acids, we adapted the method reported in[3]. First, we derivatized each freeze-extracted sample by mixing 3.5 µl of 50 mM borate buffer and 0.5 µl of each sample, then we added 10 µl of 6-aminoquinolyl-N-hydroxysuccinimidyl carbamate (AQC, S041, Synchem, USA), 4 mg/ml in anhydrous acetonitrile and mixed by pipetting. Before injection, samples were incubated for 10 min at 55 °C. Column: ACCLAIM Vanquish C18 (2.2 µm, 2.1 mm X 150 mm) (ThermoFisher, USA), Eluent A: H2O + 50 mM ammonium formate (17843, Honeywell, USA) + 0.8 % v/v formic acid (A117-50, Fisher Scientific, USA), Eluent B: Acetonitrile. With a column temperature of 45 °C and a flow of 0.65 ml/min, we measured fluorescent emission at 473 nm upon excitation at 266 nm (both with 50 Hz). We used the following method: 0.5 % B from 0 to 0.548 min, increase to 5.2 % B at 3 min, to 9.2 % B at 8.077 min, to 14 % B at 8.626 min, isocratic at 14 % B until 9.5 min, increase to 19.2 % B at 11.227 min, to 19.5 % B at 13.696 min, to 90 % B at 14.4 min and equilibration at 0.5 % B for 6 minutes. Calibrations with different solute concentrations in standard solutions were done and used for analysis. Data was analyzed using Chromeleon 7.2.10 (ThermoFisher Scientific, USA). Peak analysis was done by automatic peak integration and manually adjusted when needed.

Nucleotide detection

Ribonucleotides present in each freeze-extracted sample (1.5 µL) were mixed with 6 µL of 50 mM borate buffer and were detected by high performance liquid chromatography (HPLC) without prior nucleotide purification. The system (Vanquish Flex (VF-S01-A), ThermoFisher, USA) was equipped with a binary pump and a variable wavelength detector. For separation, we used: a Symmetry C18 column (3.5 µm pore size, 2.1 mm diameter, 150 mm length, WAT106005) (Waters, USA), Eluent A: H2O (0.1 % v/v Formic acid) and Eluent B: Acetonitrile (0.1 % v/v Formic acid). A flow of 0.3 ml/min was applied with column temperature set at 30 °C and UV absorption detection at 260 nm.

The following method was used: start with 0% B, increase to 10% at 10 minutes, further to 15% at 24 minutes, followed by a 1 minute washing step at 75% B and equilibration at 0% B for 10 minutes. Individual peaks were validated by mass spectrometry. Data was analyzed using Chromeleon 7.2.10 (ThermoFisher Scientific, USA). Peak analysis was done by automatic peak integration and manually adjusted when needed.

Fluorescence analysis

Analyses of the recorded fluorescence intensities were conducted with another custom-made LabVIEW program, which corrected for inhomogeneous illuminations. The fluorescence intensities values displayed in the figure graphs were obtained as follows: first, an area of 40x40 pixels was selected per image per position, then the average fluorescence value of each area was calculated and the background subtracted with the average signal within the same selected area from the first image acquired. The resulting values were used as a single data point per position per image acquired per time. Unless otherwise stated, total sfGFP, when mentioned in the text or figure captions, corresponds to the sum of the average values of all acquired positions per chamber considering the last time point.

Fold accumulation calculations

First, the volume of each freeze-extracted section was measured and the volume error per fraction calculated. Calculation was as follows: the ratio between the difference in volume of each fraction and the average volume obtained from the three fractions of each trap with respect to 1/3 of the total volume of the three fractions. Since the errors obtained per fraction were less than 6 % we did not take them into account for the calculations of the fold accumulation plots shown in Fig. S9A,B and C). Note that the fold accumulation was only calculated for the chambers subjected to temperature difference and thermogravitational accumulation i.e. placed in vertical orientation since only in such cases DNA, proteins and ions were accumulated. Absolute fold accumulation was calculated by first assessing the average signal (DNA band intensity, total protein lane intensity, single band intensity and ion concentration) from the three extracted fractions per chamber. The average value corresponds to the initial signal value at t=0 h before the application of the temperature difference and therefore it can be used as a reference signal from which to calculate the fold accumulation. The calculation resulted by dividing the measured values per fraction (TOP, MID and BOT) by the average value of the three fractions. The reported relative fold accumulation instead, was calculated by dividing the signal from the BOT sections to the signal from the TOP sections.

Note that for the case of the absolute fold accumulation it is not possible to obtain a value higher than 3-fold since we divided and extracted the frozen samples into 3 parts and therefore the measured and quantified signal of DNA, proteins, ribonucleotides, amino acids and ions corresponded to the average signal per fraction volume.

Note that 90 ° rotated thermophoretic chambers subjected to temperature difference did not show any effective accumulation since the frozen samples from these chambers were divided and extracted in the same way as the vertically oriented chambers i.e. from the longitudinal axis (35 mm). However in such cases thermophoresis was still active and driving the TX-TL molecular components to the cold sapphire (~27 °C).

Finite element model of the thermogravitational accumulation of sfGFP

We used the finite element software framework Comsol (version 5.4, Göttingen, Germany) to understand the complex process of thermogravitational accumulation of sfGFP within TX-TL reactions (PURExpress NEB). This software package enables the coupling of heat conduction, fluid flow, and material transport of super folder green fluorescent protein (sfGFP), allowing us to compare the simulation data to our experimental results shown in the main text and supplementary

material. First, we used the numerical model to obtain the effective thermophoretic properties of the expressed sfGFP (a). Then, we estimated the optimal thickness of the thermophoretic chambers that maximizes the thermogravitational accumulation of sfGFP and co-accumulated molecules within our experimental time scale (b). Finally, we simulated the retention of sfGFP against a flow of water validating the experimental results in Fig 2B and we extrapolated the retention effect for long, experimentally unachievable timescales (c).

(a) Determination of the thermophoretic properties of sfGFP:

To determine the thermophoretic properties of the accumulated sfGFP, we set up a simplified 2-dimensional model of the thermophoretic chamber shown in (Fig. S1B) with a thickness of 170 µm and a height of 35 mm. To couple all involved physical effects, we first solved the steady state of heat conduction:

(1) $div(k \cdot \nabla T) = Q,$

With k being the thermal conductivity (temperature dependent, 0.6 W/mK at 27°C), ∇T the temperature gradient, and Q the heat source at the hot side of the chamber. By setting the temperatures at the boundaries of the chamber to T_{hot} and T_{cold}, respectively, we obtained a linear temperature profile inside the chamber. The solution of equation (1) was then coupled to the Navier-Stokes equation:

(2) $\rho(u \cdot \nabla)u = \nabla \cdot \left[-pI + \mu(\nabla u + (\nabla u)^T) - \frac{2}{3}\mu(\nabla \cdot u)I\right] + \rho g,$

that describes the velocity of the fluid u. Hereby, I denotes the unit vector and g the gravitational acceleration. Coupling from equation 1 was implemented by the temperature-dependent density $\rho(T)$ and dynamic viscosity $\mu(T)$ of water. Equation 2 was solved via the steady state form of the continuity equation:

(3) $div(\rho \cdot u) = 0,$

leading to the full 2-dimensional velocity field $u(x,y)$ inside the thermophoretic chamber. Both, the temperature and velocity profile were then coupled to the time-dependent continuity equation that describes the motion of dilute species (e.g. sfGFP):

(4) $\frac{\delta c}{\delta t} = -div(-D\nabla c + c \cdot (u - D_T \nabla T)),$

With $c(t,x,y)$ being the concentration of sfGFP, D its diffusion coefficient and $D_T = -v_{th}/\nabla T$ its thermophoretic mobility that leads to a thermophoretic drift v_{th}. The strength of the thermophoretic effect over the counteracting diffusion is described via the Soret coefficient $S_T \equiv D_T/D$ (2). After solving equations 1-4, the concentration profile was averaged within the chamber sections corresponding to the same sections over which we performed the fluorescence analysis from the experiment shown in (Fig. 2A and Fig.S5A).

In order to estimate the Soret coefficient of sfGFP inside the complex TX-TL reaction solutions, we performed the above numerical calculations to obtain the time-dependent concentration profiles $c(t,x,y)$ for all possible combinations of the parameter sets of different temperature differences $\Delta T = \nabla T \cdot width$ (*8K*, *10K*, *12K*, *14K*, *16K*), diffusion coefficients D ($10 \cdot 10^{-12} m^2/s, 20 \cdot 10^{-12} m^2/s$, $50 \cdot 10^{-12} m^2/s$, $100 \cdot 10^{-12} m^2/s$, $150 \cdot 10^{-12} m^2/s$, $200 \cdot 10^{-12} m^2/s$), thermophoretic mobilities D_T ($3 \cdot 10^{-12} m^2/sK$, $2.5 \cdot 10^{-12} m^2/sK$, $2 \cdot 10^{-12} m^2/sK$, $1.5 \cdot 10^{-12} m^2/sK$, $1 \cdot 10^{-12} m^2/sK$, $8 \cdot 10^{-13} m^2/sK$, $6 \cdot 10^{-13} m^2/sK$, $0.4 \cdot 10^{-13} m^2/sK$, $0.2 \cdot 10^{-13} m^2/sK$) and dynamic viscosities $\mu(T)$ ($1 \cdot \mu(T)$, $1.5 \cdot \mu(T)$). The additional modeling of an increased viscosity was necessary since 1x concentrated TX-TL reactions are more viscous than water. Then, we linearly interpolated the concentration profiles $c(t,x,y)$ within this four-dimensional solution space using a custom-made LabVIEW tool that also implemented a Levenberg-Marquardt algorithm for fitting to the

experimental data shown in (Fig. 2A and Fig. S5A). Using the experimentally determined temperature difference $\Delta T = 13\ K$ (see material and methods), we obtained the Soret coefficient $S_T = 0.01\ 1/K$ (with a diffusion coefficient $D = 56 \cdot 10^{-12} m^2/s$) and 1.3-fold increased viscosity compared to the viscosity of water $\mu_{Pure}(T) = 1.3 \cdot \mu(T)$. The standard deviation of both coefficients was less than 5%. Finally, a second fitting step of the temperature difference validated the experimentally measured 13 K.

(b) Determination of the optimal thickness of the thermophoretic chambers:

The steady-state concentration profile obtained by thermogravitational accumulation usually gets steeper when decreasing the chamber thickness, when keeping constant the applied temperature difference, due to the increased temperature gradient (∇T). However, the transient accumulation process also slows down because of the strongly reduced convection speed of the liquid. For this reason, it was crucial to estimate the optimal thickness for a maximal accumulation at a given experimental time scale. On the one hand, increasing too much the chamber thickness leads to fast relaxation times of the accumulation process but generates low absolute concentrations. On the other hand, chambers that are too thin will reach steady state after months or years and are less efficient than thicker chambers reaching steady state within hours or days.

Therefore, we performed the above numerical calculation for the obtained diffusive mobilities and Soret coefficient of sfGFP ($D = 56 \cdot 10^{-12} m^2/s$, $S_T = 0.01\ 1/K$) in a two-dimensional thermophoretic chamber of the same height ($h = 35mm$), and temperature difference ($\Delta T = 13K$) and varied the thickness of the chamber between $50\ \mu m$ and $250\ \mu m$. We found out an optimal thickness of $\sim 170\ \mu m$ by examining the concentration profiles i.e. the absolute fold accumulation ($\frac{c}{c_0}$) at the lowest three positions (0 to 8.75 mm from the bottom) after 4h of thermogravitational accumulation, corresponding to the timescale when the in vitro TX-TL gene expression typically reaches steady state (Fig. S4A). Therefore, for all experiments shown in this article we used thermophoretic chambers $170\ \mu m$ thick.

(c) Retention of sfGFP against a water flow by thermograviational accumulation:

To extrapolate the retention of the accumulated sfGFP over a long period of time, we extended the above simulation by an inflow, which was coupled to the sfGFP concentration and was removed continuously at the same rate. Accordingly, equation 4 was extended by a source and sink term:

(5) $$\frac{\delta c}{\delta t} = -div\left(-D\nabla c + c \cdot (u - D_T \nabla T)\right) + R.$$

With *R* defined as:

(6) $$R = c(t,x,y) \cdot \frac{q_{flow}}{h_{flow}(q_{flow}) \cdot width \cdot thickness} + c_{in}(t,x,y),$$

with $c_{in}(t,x,y) = 0$, as the inflow in the experiment was Milli-Q water. In equation 6, q_{flow} denotes the inflow volume rate, *width=6.5 mm* and *thickness=0.17 mm,* the geometry parameters of the thermophoretic chambers, and $h_{flow}(q_{flow})$ the flow-dependent, effective height of the interaction volume. The latter value was estimated by performing a 3D simulation of the fluid flow alone, taking into account the microfluidic geometry of the experiment in (Fig. S7). This strategy led to fast simulation times, allowing us to model and extrapolate the experimental retention data shown in Figure 2B. Since the experiment from Figure 2B already starts with a fully formed accumulation profile, we also used this as the initial concentration profile with a well-fitting double-exponential model, normalized to the average sfGFP concentration of the whole thermophoretic chamber (Fig. S8A):

(7) $$c(0,x,y)/c_{avg} = A_0 + A_1 \cdot exp\left(-\frac{x-x_0}{\tau_1}\right) + A_2 \cdot exp\left(\frac{x-x_0}{\tau}\right)$$

Hereby the fitting coefficients were $A_0 = -0.04 \pm 0.02$, $A_1 = 0.87 \pm 0.07$, $\tau_1 = 2.6 \pm 0.2$, $A_2 = 2.36 \pm 0.05$, and $\tau_2 = 14.0 \pm 0.6$. Due to the extreme concentration differences at this starting condition, we had to include a potential concentration dependence of the Soret coefficient S_T, as previously discussed in[4]. Accordingly, we scaled the Soret coefficient with the Debye-Hückel length $\lambda_{DB} \sim 1/\sqrt{c(t,x,y)}$, which assumes that the salt ions inside the TX-TL reactions are co-accumulated with the charged macromolecules.

Additionally, we had to consider that at the start of the experiment, i.e. before the start of the water flow, the viscosity of the solution was closer to that of water in the top partition of the chamber due to the strong thermophoretic depletion of sfGFP. We took this into account within our model by assuming a concentration-dependent viscosity $\mu'(T, c(t,x,y))$. We estimated this value by a separate measurement, tracking the Brownian motion of 2 µm polysterene beads (L4530, Sigma) at a 26 °C for various dilutions of TX-TL solutions (Fig. S8B). The new viscosity model fitted well to a power law

(8) $\mu'(T, c(t,x,y)) = \mu(T) \cdot \alpha(c(t,x,y)) = \mu(T) \cdot (1 + A \cdot c(t,x,y)^p)$,

With $A = 0.36 \pm 0.04$ and $p = 0.9 \pm 0.1$.

Note that in Figure 2B we fitted the flow rate to 1.5 nL/s (5.4 µL/h) which lies well within the error range of the complex microfluidic system that was experimentally set to 3 nL/s (10.8 µL/h).

(d) Phosphate waste removal under a water flow regime

Phosphate accumulation and diffusion kinetics were added to the model alongside sfGFP to simulate waste removal under gradient or isothermal conditions. An exponential equation was used to fit the experimental data.

(9) $c_{phos}(0,x,y)/c_{phos\ avg} = A_0 + A_1 \cdot exp\left(-\frac{x}{\tau}\right)$

With A_0 = 0.02, A_1 = 46 and τ = 4.7. The Soret and Diffusion coefficient values were obtained from a previous study[5] ($D = 9.59 \cdot 10^{-10} m^2/s$, $S_T = 0.7 \cdot 10^{-3} 1/K$). The phosphate was also subjected to the same conditions as the sfGFP for this simulation including the dynamic viscosity (8) (determined by the concentration of sfGFP) and the outflow rate (6).

(e) ATP accumulation and conversion to phosphate

To further determine the reach of the model, we simulated a flow of ATP at constant concentrations and the conversion to phosphate. The known Diffusion coefficient[6] $D = 7 \cdot 10^{-10} m^2/s$ was used to calculate the Soret coefficient $S_T = 0.079317\ 1/K$ following the same protocol as for sfGFP. The conversion rate R_{ATP} was simulated using a Michaelis-Menten equation based on the activity of T7 polymerase[7] (10). In this reaction ATP is incorporated into the growing RNA strand with the production of the waste molecule phosphate.

(10) $R_{ATP} = \frac{k_{pol} \cdot c(t,x,y) \cdot c_{pol} c_{ATP}(t,x,y)}{K_m + c_{ATP}(t,x,y)}$

Where R_{ATP} is the rate at which ATP is consumed and Phosphate is produced, k_{pol} = 222 1/s maximum incorporation rate of ATP. c_{ATP}(t,x,y) is the concentration of ATP inside the simulated chamber and K_m = 0.076 mol/m^3 is the equilibrium dissociation constant of ATP. c_{pol} = 0.1016 mol/m^3 is the concentration of the T7 polymerase typically present in an undiluted PURE reaction[8]. For this case c(t,x,y) was set to be unitless and used as a scaling factor for c_{pol} to account for accumulation/depletion throughout the chamber.

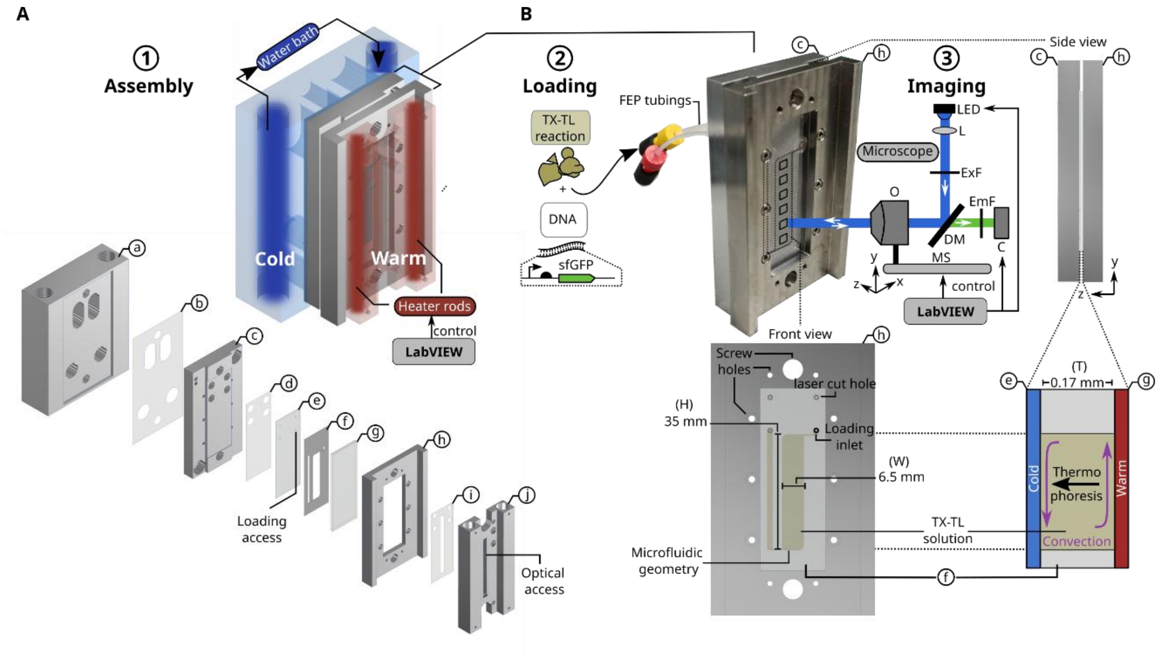

Fig. S1. General overview of the experimental setup. (A) A detailed schematic illustration of a thermophoretic chamber. The assembly [1] is described in detail in the material and methods. Shown are all the component parts of a chamber. (a) aluminum support, (c) aluminum base, (e) back sapphire plate (cold side), (f) Teflon FEP layer with microfluidic geometry, (g) front sapphire plate (warm side), (h) steel frame support, (j) aluminum support for heater rods with a rectangular slit that allows for optical access and (b,d and i) heat conducting foils added to favor heat conductivity among the different surface components. (a-e parts are perforated with holes to allow access for loading. Temperature differences are applied by heating (j) with electrical heater rods and by actively cooling down (a) via a water bath. (a and j) are shown partially transparent in the assembled chamber to highlight the water flow (dark blue) and heater rods (red). **(B)** Top left: picture of a partially assembled thermophoretic chamber ready to use for the experiments. (a) and (j) are not shown to allow the visualization of the microfluidic geometry (dotted structure) and FEP tubings. Loading [2]: TX-TL reaction solutions and DNA are loaded inside the chamber via Teflon FEP tubings through a small inlet on the back sapphire plate (see material and methods). Imaging [3]: A custom made microscope mounted in a motorized stage (MS) that moves in three directions in space (x,y,z) sequentially acquires the fluorescence arising from the TX-TL solutions at different positions of the chamber (black squares). The microscope is equipped with an objective (TL2x-SAP, Thorlabs), two light emitting diodes (LED) (470, 625 nm) and a camera (Stingray 145-B, Allied Vision). However, for simplicity reasons, the figure only shows the relevant optical components to monitor the fluorescence of sfGFP. i.e. dichroic mirror (ZT488/561rpc, F53-495 AHF), excitation (ExF) (D470/40x, Chroma) and emission (EmF) (FELH0500, Thorlabs) filters, a lens (L) that collimates the light from an LED (470nm, M470L2, Thorlabs). A custom-made LabVIEW program controls the temperature of the heater rods, the move of the motorized stage of the microscope and the imaging acquisition. Top right: schematic of the side view of the chamber. Bottom left: front view of the chamber from which the microfluidic geometry used for the thermogravitational accumulation experiments can be seen in detail. Displayed are the height (H) and width (W) of the central column where the TX-TL solution is loaded. Four laser-cut holes allow back access to the chamber. (Highlighted in black, the inlet hole from which the TX-TL sample is loaded). Six screw holes on the sides help fix the steel frame to the aluminum base, 4 screw holes (two on top and two at the bottom) help fix the aluminum support for the heater rods to the steel frame and finally two screw holes top, bottom help fix the chamber to the aluminum support. Bottom right: enlarged side view schematic representation of the back cold sapphire (blue)-FEP-front warm sapphire (red) with a thickness (T) of 0.17 mm. When a temperature difference is applied, thermophoresis and convection combined drive the thermogravitational accumulation of the components of the TX-TL solution.

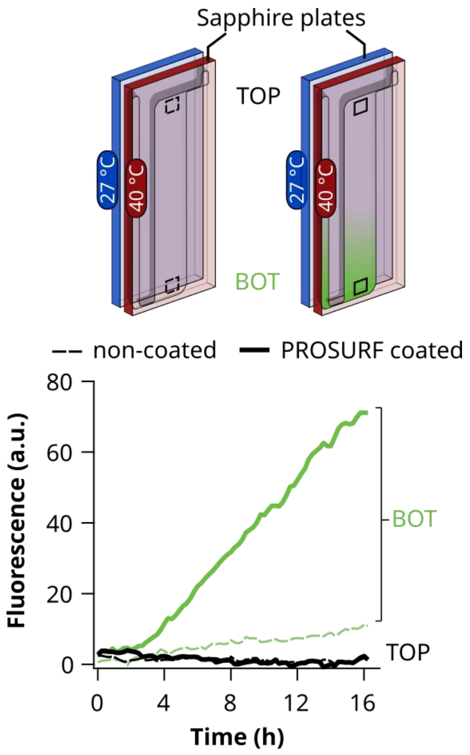

Fig. S2. Gene expression test in PROSURF coated versus uncoated thermophoretic chambers. The walls of thermophoretic chambers i.e. sapphire plates were either coated with PROSURF (see material and methods) or non-coated to test for sfGFP expression recovery under a temperature difference (27-40 °C). The graph shows the fluorescence kinetics of sfGFP expression from 0.33x concentrated TX-TL reactions measured at the top and bottom positions (black squares). Solid and dashed lines correspond to the fluorescence acquired from the coated and uncoated chambers respectively. Green and black lines correspond instead to the bottom and top positions of the chambers respectively.

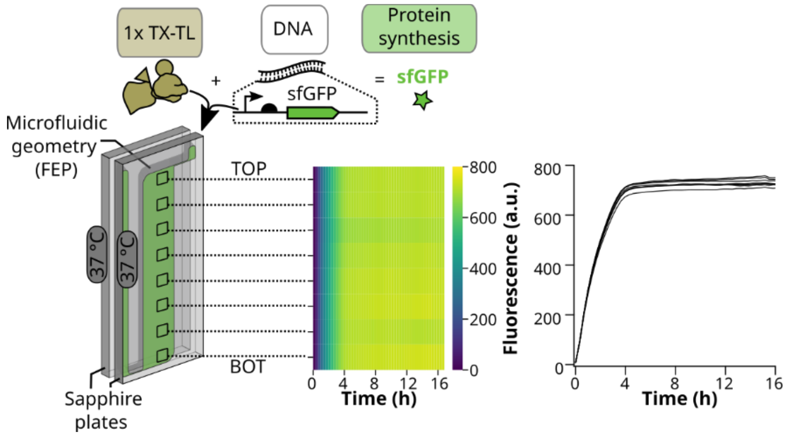

Fig. S3. sfGFP in vitro protein synthesis works inside thermophoretic chambers incubated isothermally. To assess whether the surfaces of the thermophoretic chambers i.e. Teflon FEP and sapphire were compatible with cell-free gene expression, a 1x concentrated TX-TL reaction was supplemented with 5.9 nM linear PCR DNA template coding for sfGFP. The reaction was incubated at 37 °C for 16 h and sfGFP fluorescence monitored at 8 different positions (black squares) with a custom made motorized microscope (Fig. S1B). Fluorescence analysis was performed with a custom LabVIEW analysis program (see material and methods). To visualize the homogenous protein expression along the chamber, recorded fluorescence values are displayed in a heatmap plot and a line graph. Each line from the graph corresponds to the fluorescence signal analyzed at each position.

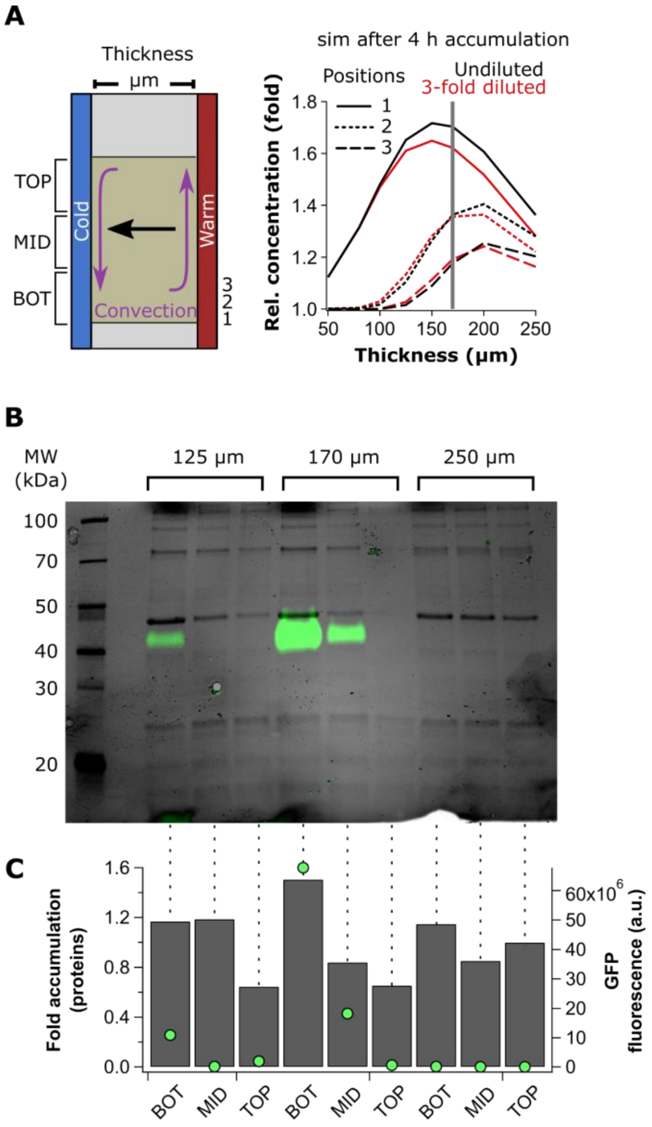

Fig. S4. Assessing the optimal thickness of the thermophoretic chamber using finite element simulations. The thickness of the thermophoretic chambers was optimized to maximize the thermogravitational accumulation of sfGFP within the time scale of the TX-TL solution before reaching steady state (see materials and methods). **(A)** The relative concentration of sfGFP after 4 hours of simulated thermogravitational accumulation in a 2D chamber subjected to ΔT= 13 K and with a height = 35 mm is plotted against different chamber thicknesses. A thickness of 170 µm was chosen, which corresponded to the optimal average chamber thickness from the three bottommost positions (1 to 3, ranging from 0 mm to 8.75 mm). A similar accumulation profile was observed for both diluted and undiluted TX-TLsolutions. **(B)** Denaturing SDS polyacrylamide gel showing bottom, middle and top fractions of the three chambers of different thicknesses. Green bands indicate the presence of sfGFP. Black bands correspond to the proteins of 3-fold diluted PURExpress (NEB) **(C)** Gel band analysis confirmed that sfGFP was synthesized most abundantly in the chamber with a thickness of 170 µm at the bottom and middle fractions while 125 µm shows a signal only in the bottom fraction. At a thickness of 250 µm shows no accumulation and therefore no expression could be observed.

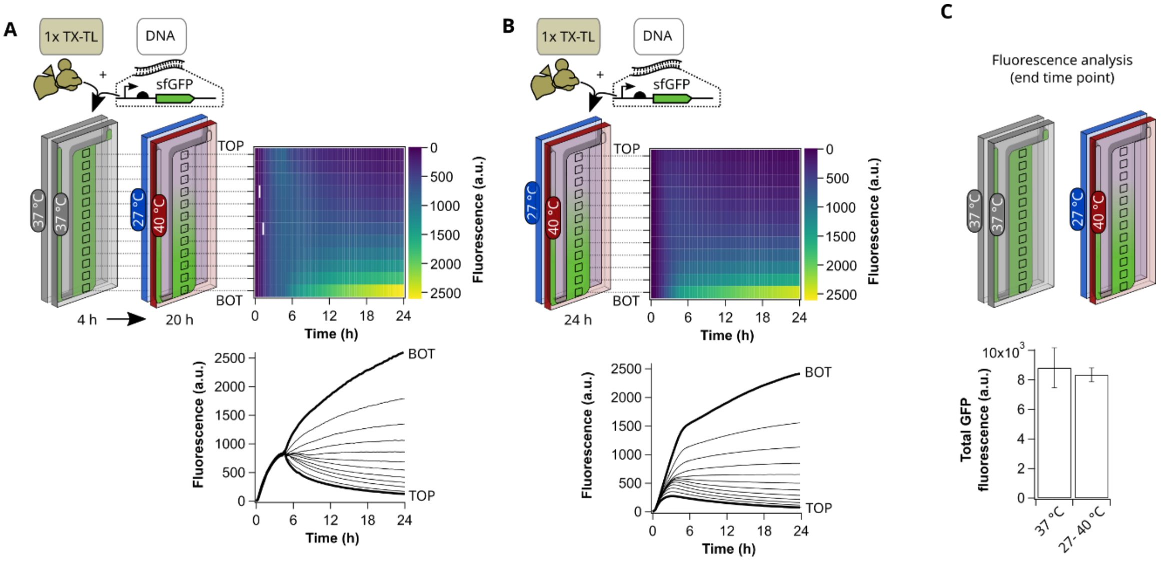

Fig. S5. Temperature differences across thermophoretic chambers do not impair in vitro gene expression and accumulate sfGFP inside thermophoretic chambers. 1x concentrated TX-TL reactions were assembled with 5.9 nM of linear PCR fragment coding for sfGFP and loaded inside thermophoretic chambers that were incubated isothermally and/or subjected to a temperature difference to assess for in vitro gene expression and accumulation. The fluorescence signal from in vitro expressed sfGFP was acquired over time at 12 different positions (black squares) along the vertical axis of the chambers with a custom-made fluorescence microscope. **(A)** Heat map and graph lines showing the fluorescence kinetics of sfGFP when initially incubated at 37 °C for 4 h to allow the in vitro expression of the protein and subsequently subjected to (~ 27-40 °C) for 20 h to allow for sfGFP accumulation. Note that the fluorescence kinetics from 4 to 20 h correspond to the same values shown in Fig. 2A. For simplicity reasons in Fig. 2A the fluorescence values from six out of twelve positions were plotted. **(B)** Heat map and graph lines showing the fluorescence kinetics of sfGFP when subjected to (~ 27-40 °C) during the entire incubation time. For panels (A, B) the most top and bottom positions from which the fluorescence had been recorded are highlighted in bold. **(C)** Total sfGFP fluorescence indirectly indicates the yield of sfGFP when synthesized under a temperature difference or incubated isothermally. The displayed average values in the bar plot corresponds to the sum of the end time point values along the 12 different positions (corresponding to 24 h for thermophoretic chambers subjected to temperature differences or 16 h for chambers incubated isothermally at 37 °C). Error bars correspond to ±SD, n=3.

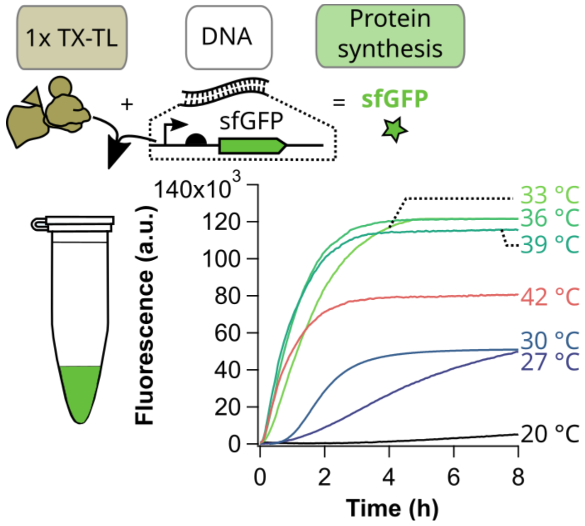

Fig. S6. Temperature dependent sfGFP expression kinetics in test tubes. 1x concentrated TX-TL reaction solutions were supplemented with 5.9 nM linear PCR DNA template coding for sfGFP. 4 µL reactions were incubated at different temperatures inside test vials for 8 h. Fluorescence signal was acquired with the FAM channel in a qTower^3 (Analytik Jena) thermal cycler. Optimal temperature ranges from 33-39 °C. Working temperature ranges from 27-42°C.

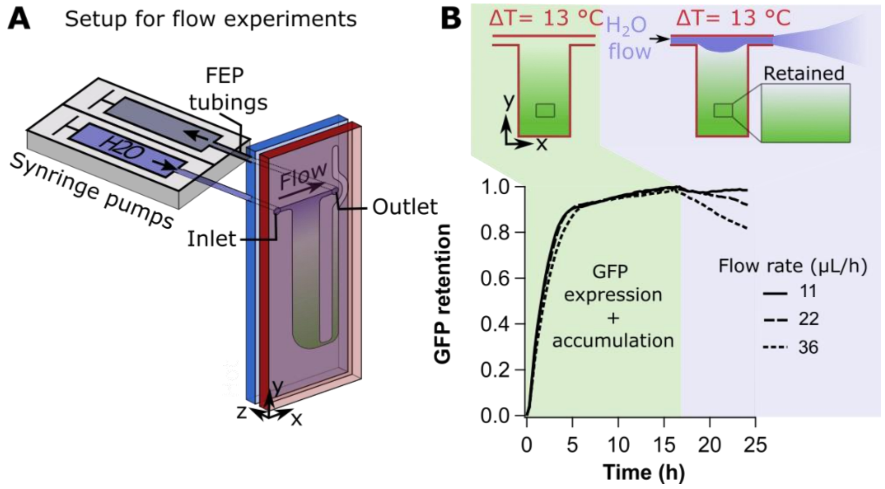

Fig. S7. Temperature differences across thermophoretic chambers retain and protect sfGFP against dilution. Left: Schematic of the setup used for the experiments shown in the right panel and Fig. 2B and 2C. The microfluidic geometry was slightly modified to allow for water or feeding solution flow through a thin channel (1 mm Ø) located at the top of the thermophoretic chamber. The flow was controlled by two syringe pumps (nMESYS) connected via FEP tubings (1 mm inner Ø) to the chamber inlet and outlet. Right: Thermophoretic chambers subjected to a temperature difference (ΔT=13 °C, warm ~ 40 °C-cold ~ 27 °C), expressed and accumulated sfGFP over 16h of incubation. Then, Milli-Q water was pushed through the upper channel of the chambers to assess the retention of sfGFP provided by the temperature difference. The graph shows the total sfGFP remaining over time inside chambers subjected to 11, 22 and 36 µL/h water flow rates, normalized against the maximum value of fluorescence obtained after 16 h of incubation prior the start of the flow.

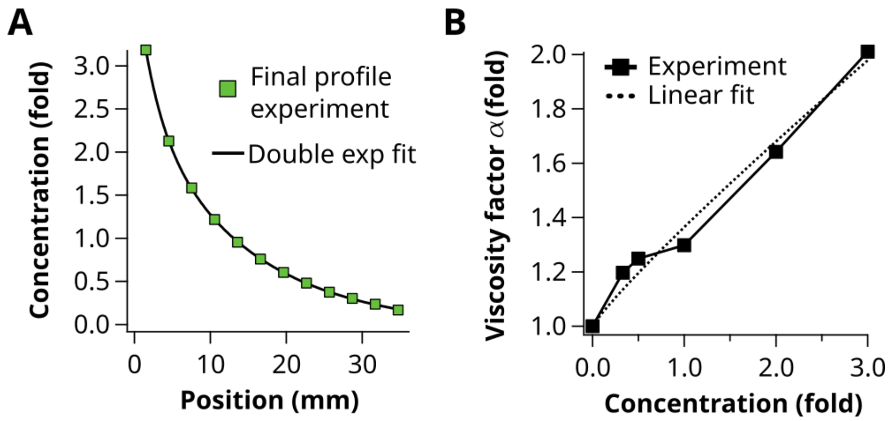

Fig. S8. Estimation of model parameters for sfGFP retention against a flow of water. (A) The end time point accumulation profile of pre-expressed and accumulated sfGFP (green boxes) from Figure 2A and S5A was fitted by a double exponential model (solid line), equation 7 in material and methods. The double exponential model was then used as the initial concentration profile of sfGFP before starting the flow of water. **(B)** A concentration dependent viscosity μ was considered in the finite element simulation to better model the retention of sfGFP under a flow of water. The concentration dependent viscosity μ scales with a prefactor α, that fits well to a power law (dashed line), see equation 8 in material and methods. Experimental viscosity values (black boxes) were obtained by tracking polystyrene beads at different TX-TL solution concentrations.

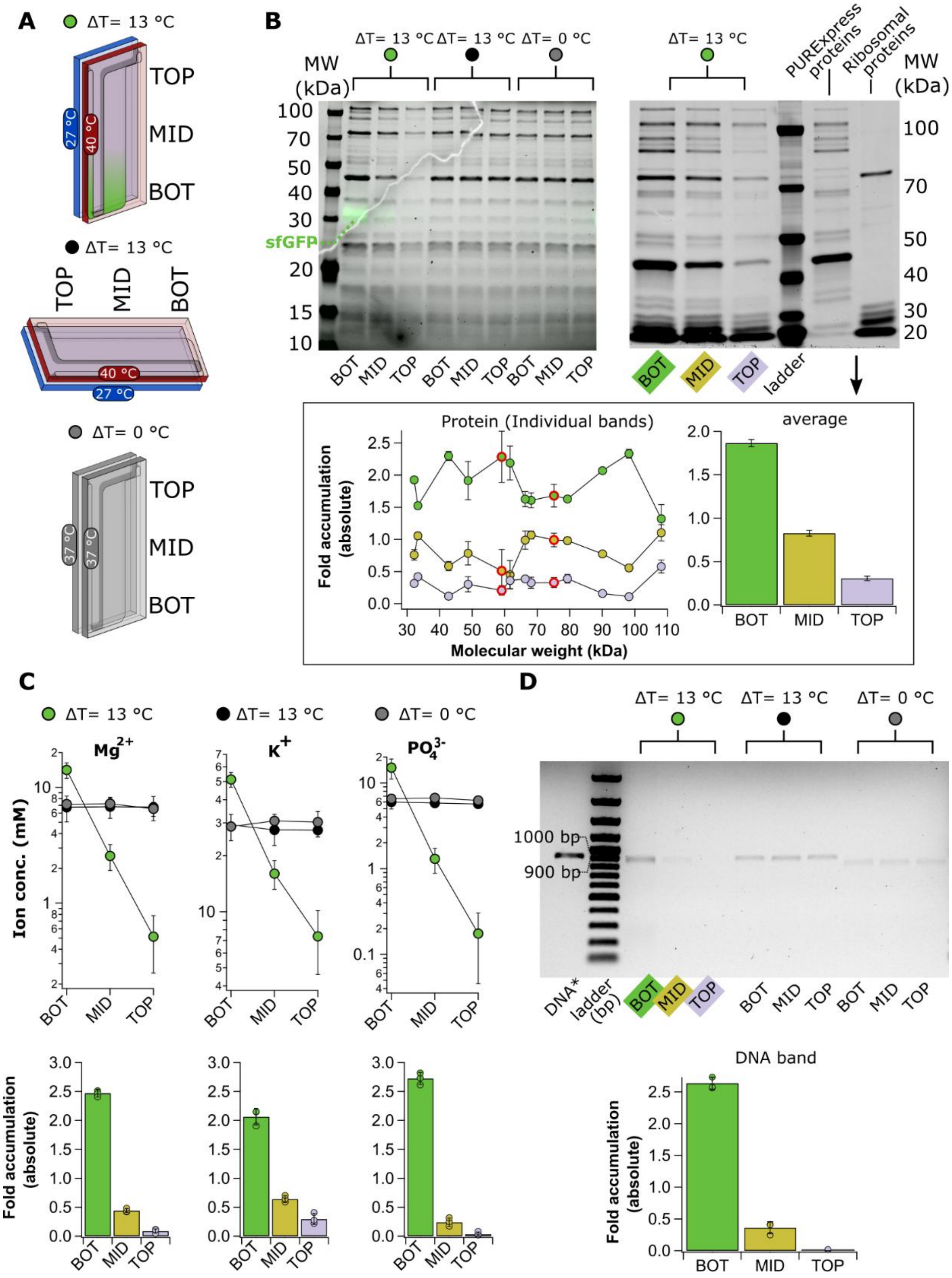
A
ΔT= 13 °C
TOP
MID
BOT
27 °C
40 °C
ΔT= 13 °C
TOP
MID
BOT
40 °C
27 °C
ΔT= 0 °C
TOP
MID
BOT
37 °C
37 °C
B
ΔT= 13 °C
ΔT= 13 °C
ΔT= 0 °C
MW (kDa)
100
70
50
40
30
sfGFP
20
15
10
BOT MID TOP BOT MID TOP BOT MID TOP
ΔT= 13 °C
PURExpress proteins
Ribosomal proteins
MW (kDa)
100
70
50
40
30
20
BOT MID TOP ladder
Protein (Individual bands)
Fold accumulation (absolute)
Molecular weight (kDa)
average
BOT MID TOP
C
ΔT= 13 °C
ΔT= 13 °C
ΔT= 0 °C
Mg2+
K+
PO43-
Ion conc. (mM)
BOT MID TOP
Fold accumulation (absolute)
BOT MID TOP
D
ΔT= 13 °C
ΔT= 13 °C
ΔT= 0 °C
1000 bp
900 bp
DNA*
ladder (bp)
BOT MID TOP BOT MID TOP BOT MID TOP
DNA band
Fold accumulation (absolute)
BOT MID TOP

Fig. S9. Accumulation of ions, DNA and proteins and determination of absolute accumulation ratios. (A) After 16 h of incubation, thermophoretic chambers filled with 0.33x concentrated TX-TL reactions were frozen and sliced into three parts (TOP, MID, BOT). Each section was prepared and subjected to DNA, protein gel electrophoresis and ion chromatography to determine the accumulation of DNA, proteins and ions. Negative controls devoid of accumulation included chambers incubated with a temperature difference (ΔT= 13 °C) rotated 90° into a horizontal orientation to stop convection (middle) and chambers placed vertically, incubated isothermally at 37 °C (ΔT= 0 °C) (bottom). **(B)** Representative images of denaturing SDS polyacrylamide gels showing the protein content distribution along the chambers. Left: merged image gel where green bands correspond to sfGFP while black bands correspond to PURExpress and ribosomal proteins visualized with stain-free technology (see material and methods). Right: image of a gel stained with SYPRO ruby (Sigma) and used to increase the separation of bands for individual band quantification. The absolute fold accumulation graph for individual bands mostly shows protein bands from PURExpress proteins that do not overlap with ribosomal proteins. Overlapping bands are highlighted in red. The absolute fold accumulation was only plotted for the vertically placed chambers incubated with a temperature difference since negative controls did not show any considerable effective enrichment in any fraction. The data is represented as the mean value of triplicate experiments ± SD. **(C)** Concentration profile along the thermophoretic chambers for Mg^{2+}, K^{+} and PO_4^{3-} ions. The data is represented as the mean value of triplicate experiments ± SD. **(D)** Agarose gel showing the DNA distribution along the thermophoretic chambers. The data is represented as the mean value of triplicate experiments ± SD.

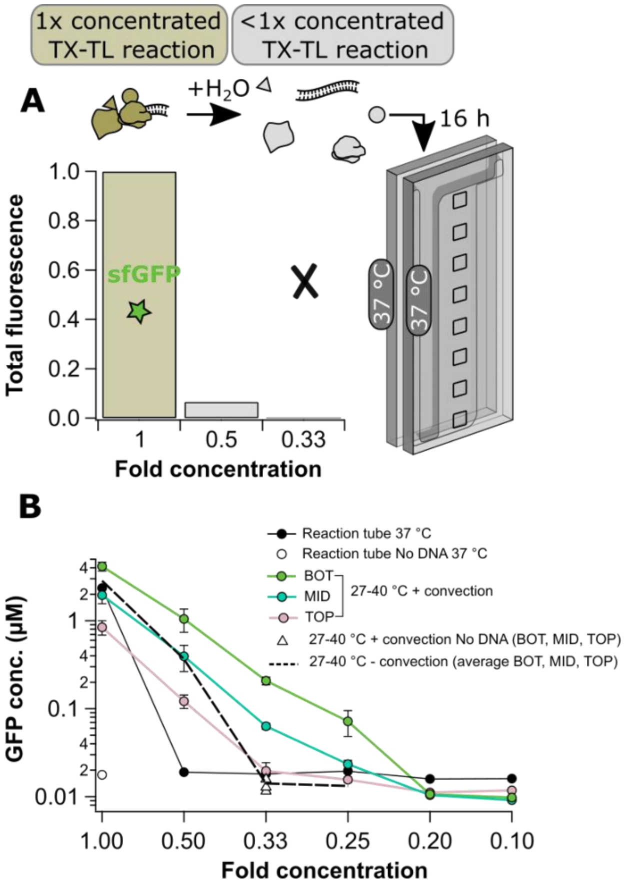

Fig. S10. Assessing in vitro sfGFP expression from diluted TX-TL reactions inside thermophoretic chambers incubated under isothermal conditions. (A) 1x concentrated TX-TL reactions were diluted 2- and 3-fold with Milli-Q water before incubation at 37 °C inside thermophoretic chambers for 16 h. To assess the fluorescent protein levels produced per reaction the fluorescence was measured with a custom-made microscope at 8 different positions along the vertical axis of the chambers (black squares). Plotted total fluorescence values (see material and methods) are normalized in this case to the sum of the fluorescence average value per position of all positions obtained from the chamber with a 1x concentrated TX-TL reaction. **(B)** sfGFP concentration yield obtained from TX-TL reactions of increasing dilutions subjected to different thermal conditions and incubated inside reaction tubes or thermophoretic chambers. sfGFP was not detected from 5x and 10x diluted TX-TL reactions. BOT, MID, TOP refer to samples extracted from thermophoretic chambers from the bottom middle and top positions. "+ convection" and "-convection" refer to thermophoretic chambers subjected to a temperature difference (27-40 °C) placed in vertical orientation with convection or rotated 90 ° into a horizontal orientation without convection respectively. No DNA indicates the absence of DNA and corresponds to the non template controls of the TX-TL reactions. Average BOT, MID, TOP is the average concentration value obtained for the three fractions. Error bars correspond to ±SD, n=3 separate experiments.

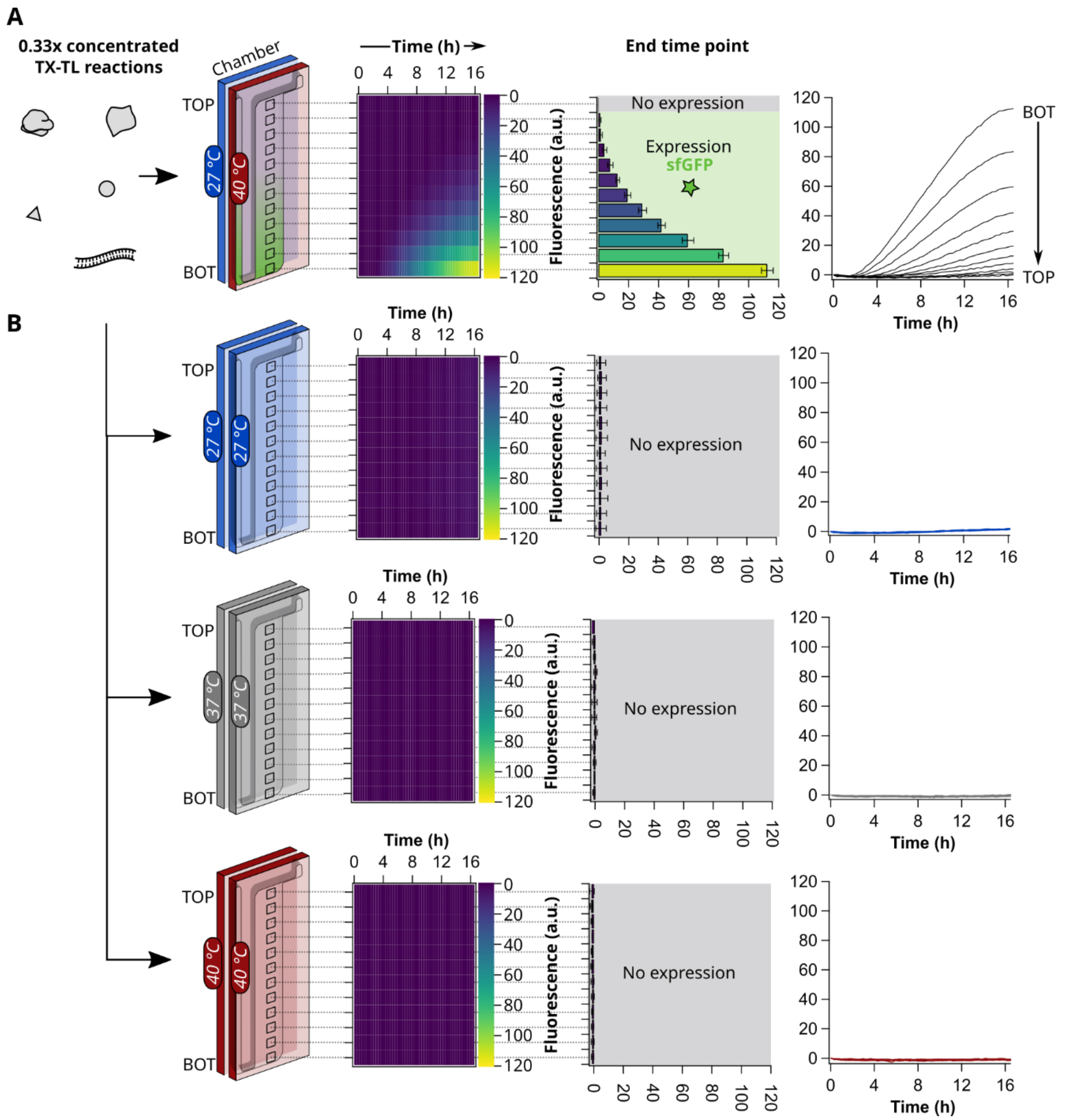

Fig. S11. sfGFP expression recovery from inactive 0.33x concentrated TX-TL reactions. 1x concentrated TX-TL reactions were diluted 3-fold with water and incubated for 16 h inside thermophoretic chambers subjected to different temperatures. To check for sfGFP expression recovery, the fluorescence arising from the chambers was acquired with a custom-made microscope at different positions along the vertical axis (black squares). Fluorescence values per position over the entire course of the experiment are displayed as heat maps plots. End time point fluorescence values are plotted as category plots. The right graphs display the same average fluorescence values as the heat maps to help visualize the kinetics of expression per condition. **(A)** 0.33x concentrated TX-TL reaction subjected to 27-40 °C gradient reactivates sfGFP expression after ~ 4h of incubation. The expressed fluorescent protein follows an exponential distribution along the vertical axis of the chamber with negligible signal at the top positions. **(B)** Negative controls devoid of accumulation and therefore not expressing sfGFP were chambers incubated isothermally at 27 °C (blue chamber), 37 °C (gray chamber) and 40 °C (red chamber). Error bars correspond to ±SD, n=3.

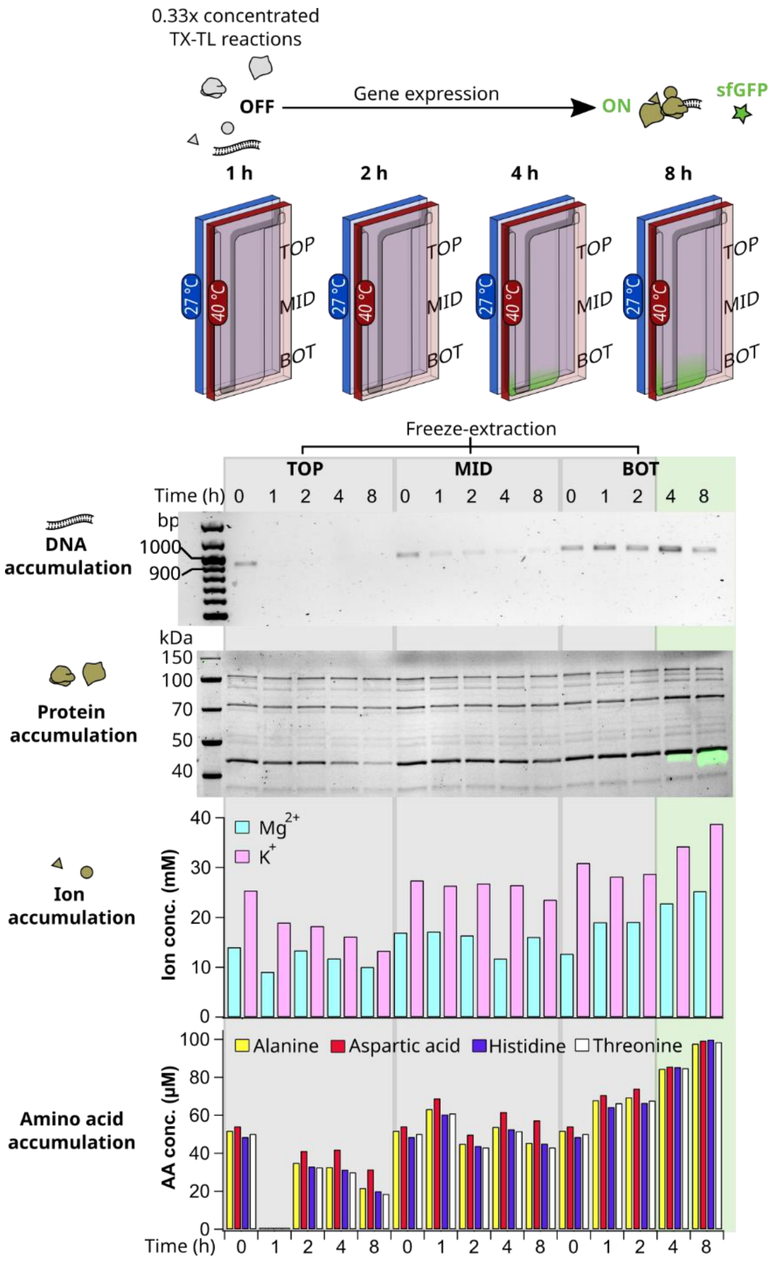

Fig. S12. Accumulation kinetics of DNA, proteins, ions and amino acids. 0.33x TX-TL solutions were incubated inside a thermophoretic chamber subjected to a temperature difference for 0,1,2,4 and 8 h. Top, middle and bottom sections were freeze-extracted and analyzed by gel electrophoresis or chromatography to assess the time course accumulation of DNA, proteins, ions and amino acids. (DNA panel) agarose gel showing the fast depletion and accumulation of DNA at the top and bottom of the chamber respectively. (Protein panel) cropped image of a denaturing polyacrylamide gel showing the onset of sfGFP expression (green bands) after 4 h of incubation, and the accumulation of TX-TL proteins (black bands). (Ion panel) bar plot showing the gradual increase in concentration of Mg^{2+} and K^+ at the bottom of the chamber. (Amino acid panel) bar plot showing the gradual increase in concentration of L-Alanine, L-Aspartic acid, L-Histidine and L-Threonine at the bottom of the chamber.

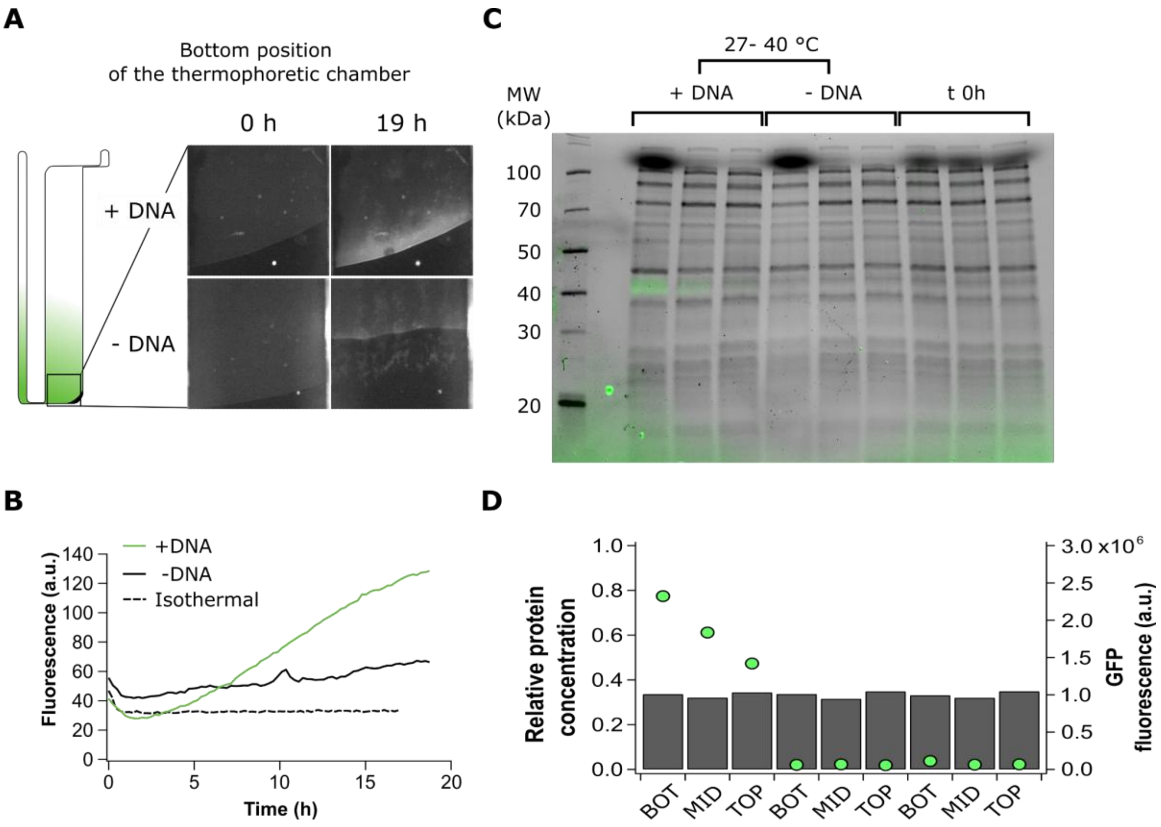

Fig. S13. Synthesis and accumulation of sfGFP from diluted cell lysate in a thermophoretic chamber. 3-fold diluted *E. coli* cell lysate reactions with and without DNA were loaded in thermophoretic chambers and subjected to a temperature difference for 19h. As a control without molecular accumulation nor protein synthesis an additional reaction was loaded in a separate chamber and frozen immediately without incubation. The chambers were frozen and samples extracted into 3 fractions (BOT, MID, TOP). **(A)** Microscopy images showing the fluorescent signal at the bottom of the thermophoretic chamber before and after incubation. The observed fluorescence is a combination of sfGFP and background signals as the solution without DNA also showed fluorescence. **(B)** Fluorescence kinetics at the bottom of the chambers with and without DNA incubated with a temperature difference and with DNA in isothermal conditions (37°C). **(C)** Denaturing SDS polyacrylamide gel showing bottom (BOT), middle (MID) and top (TOP) fractions of the three chambers. Green bands indicate the presence of sfGFP, black bands correspond to the proteins of the cell lysate. **(D)** Gel band analysis revealed that sfGFP was synthesized and was more abundant at the bottom position than at the top only in the thermophoretic chamber containing DNA and subjected to a temperature difference. Instead, the proteins in all three conditions do not follow the accumulation pattern observed for the TX-TL experiments using PURExpress (NEB).

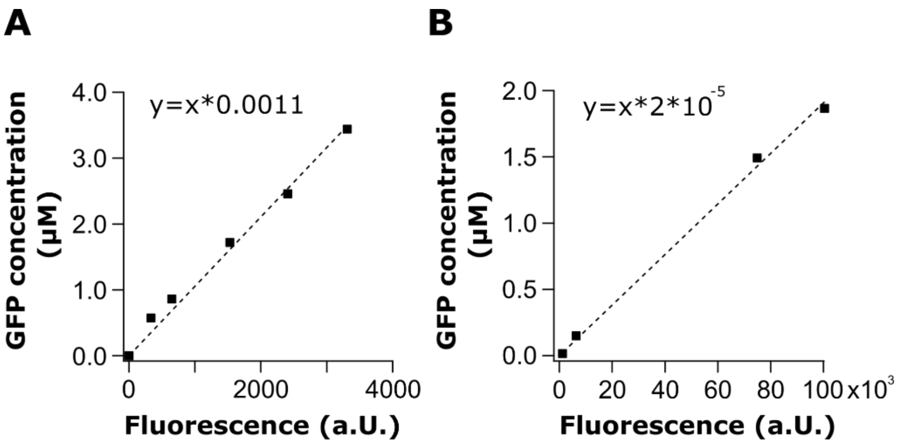

Fig. S14. Calibration curves used for converting fluorescence units into sfGFP concentrations. To calculate sfGFP concentration from fluorescence values, the fluorescence of solutions containing purified sfGFP with known concentrations was analyzed using the same methods as for the TX-TL reactions. **(A)** To assess the concentration of sfGFP for experiments shown in Fig. 2C and 3E the solutions were imaged inside thermophoretic chambers subjected to isothermal conditions at 26, 37 and 42 °C. The resulting fluorescence values for each temperature were averaged and fitted. **(B)** To assess the concentration of sfGFP for the experiment in Fig. S10B, the fluorescence at different concentrations was measured in a qTower^3 (Analytik Jena) thermal cycler in the same way as for the end time point experiments.

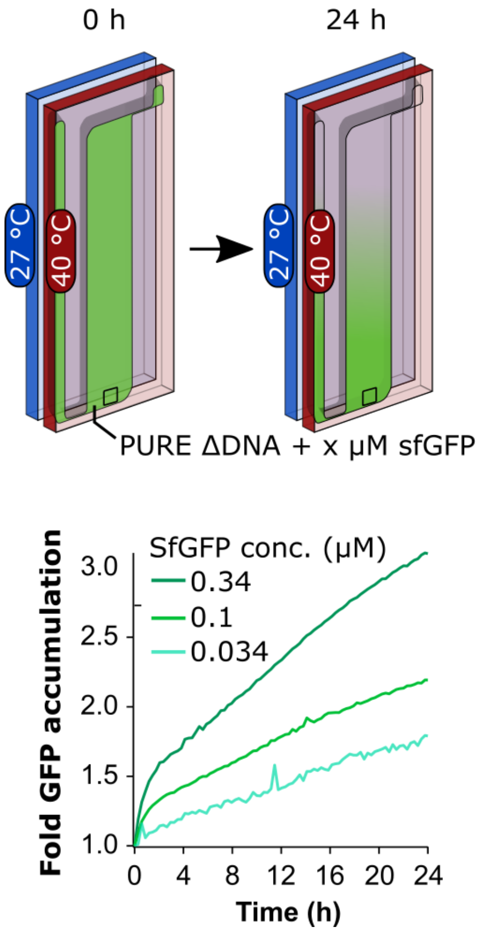

Fig. S15. Accumulation of different concentrations of purified sfGFP. 3-fold diluted PURE reactions containing no template DNA were mixed with different amounts of purified sfGFP and incubated under a thermal gradient of 13K for 24h. (top) Graphical representation of the thermal chamber containing sfGFP. Initially the sfGFP is evenly distributed throughout the chamber, however after the incubation period a clear increase in signal is detected at the bottom. (bottom) Fold increase in sfGFP signal normalized to initial concentration and plotted against time. The different shades of green denote individual sfGFP starting concentrations.

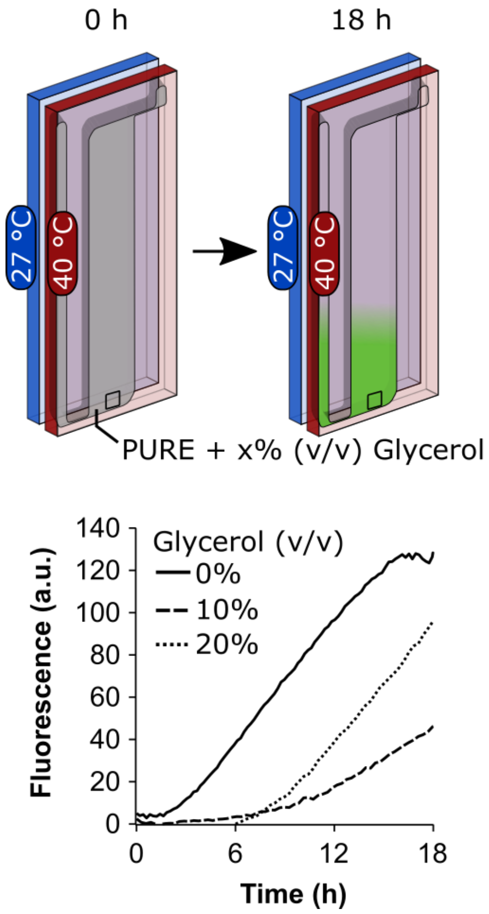

Fig. S16. Synthesis and accumulation of sfGFP in PURE reactions with increased viscosities. 3-fold diluted PURE reactions were mixed with additional amounts of Glycerol to a final concentration of 10% or 20% respectively. (top) graphical representation of the experiment. After filling the traps, the reactions were run for 18h with a temperature difference of 13K across the depth of the chamber. (bottom) Fluorescence intensity of sfGFP plotted over time for the reactions mixed with glycerol together with a control reaction where no additional glycerol was added.

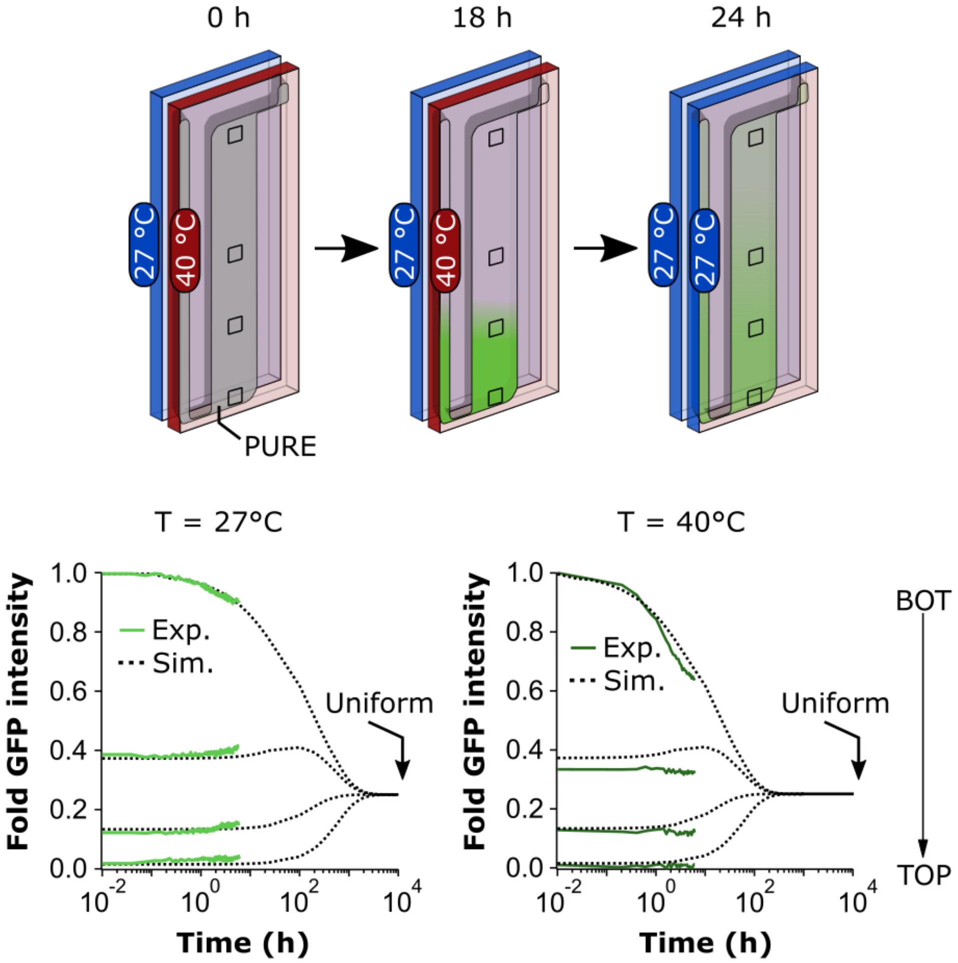

Fig. S17. Diffusion of sfGFP after synthesis at different isothermal temperatures. 3-fold diluted PURE reactions were accumulated for 18h with a temperature difference of 13K. Subsequently the temperatures on the front and back sapphires were set to 27 or 40°C respectively for another 6h in order to allow the synthesized sfGFP to diffuse. (top) graphical representation of the experiment. (bottom, solid lines) Fold decrease in sfGFP intensity at different positions in the trap normalized to the intensity of the fluorescence just before the removal of the gradient plotted against time. (dotted lines) simulated diffusion of sfGFP at 27°C for a period of 10^4 h. Uniform distribution is reached after 3000h at 27°C. For T = 40°C simulation data was adjusted timewise to correspond to the decrease in intensity at the bottom position.

	Absolute fold accumulation (TOP)	Absolute fold accumulation (MID)	Absolute fold accumulation (BOT)	Relative fold accumulation (BOT/TOP)
**Alanine**	0.133	0.521	2.346	17.6
**Aspartic acid**	0.226	0.836	1.938	8.6
**Histidine**	0.104	0.407	2.489	23.9
**Threonine**	0.090	0.361	2.549	28.2
**ATP**	0.002	0.050	2.948	1250.1
**GTP**	0.016	0.122	2.862	180.6
**UTP**	0.012	0.132	2.857	243.9

Table S1. Thermogravitational accumulation of amino acids and ribonucleotides. Reported values correspond to the absolute and relative fold accumulation of four proteinogenic L-amino acids and three ribonucleotide triphosphates.

Name	Plasmid sequence	PCR primers
pGEMT_MS2_sfGFP	AAAAGGATCTTCACCTAGATCCTTTTAAATTAAAAATGAAGTTTTAAATCAATCTAAAGTA<br>TATATGAGTAAACTTGGTCTGACAGTTACCAATGCTTAATCAGTGAGGCACCTATCTCA<br>GCGATCTGTCTATTTCGTTCATCCATAGTTGCCTGACTCCCCGTCGTGTAGATAACTAC<br>GATACGGGAGGGCTTACCATCTGGCCCCAGTGCTGCAATGATACCGCGAGACCCACG<br>CTCACCGGCTCCAGATTTATCAGCAATAAACCAGCCAGCCGGAAGGGCCGAGCGCAG<br>AAGTGGTCCTGCAACTTTATCCGCCTCCATCCAGTCTATTAATTGTTGCCGGGAAGCTA<br>GAGTAAGTAGTTCGCCAGTTAATAGTTTGCGCAACGTTGTTGCCATTGCTACAGGCATC<br>GTGGTGTCACGCTCGTCGTTTGGTATGGCTTCATTCAGCTCCGGTTCCCAACGATCAA<br>GGCGAGTTACATGATCCCCCATGTTGTGCAAAAAAGCGGTTAGCTCCTTCGGTCCTCC<br>GATCGTTGTCAGAAGTAAGTTGGCCGCAGTGTTATCACTCATGGTTATGGCAGCACTG<br>CATAATTCTCTTACTGTCATGCCATCCGTAAGATGCTTTTCTGTGACTGGTGAGTACTCA<br>ACCAAGTCATTCTGAGAATAGTGTATGCGGCGACCGAGTTGCTCTTGCCCGGCGTCAA<br>TACGGGATAATACCGCGCCACATAGCAGAACTTTAAAAGTGCTCATCATTGGAAAACGT<br>TCTTCGGGGCGAAAACTCTCAAGGATCTTACCGCTGTTGAGATCCAGTTCGATGTAACC<br>CACTCGTGCACCCAACTGATCTTCAGCATCTTTTACTTTCACCAGCGTTTCTGGGTGAG<br>CAAAAACAGGAAGGCAAAATGCCGCAAAAAAGGGAATAAGGGCGACACGGAAATGTTG<br>AATACTCATACTCTTCCTTTTTCAATATTATTGAAGCATTTATCAGGGTTATTGTCTCATG<br>AGCGGATACATATTTGAATGTATTTAGAAAAATAAACAAATAGGGGTTCCGCGCACATTT<br>CCCCGAAAAGTGCCACCTGATGCGGTGTGAAATACCGCACAGATGCGTAAGGAGAAAA<br>TACCGCATCAGGGGGTGGGACCCCTTTCGGGGTCCTGCTCAACTTCCTGTCGAGCTAA<br>TGCCATTTTTAATGTCTTTAGCGAGACGCTACCATGGCTATCGCTGTAGGTAGCCGGAA<br>TTCCATTCCTAGGAGGTTTGGGCTTAAGTATAAGGAGGAAAAAATATGAGCAAAGGAGA<br>AGAACTTTTCACTGGAGTTGTCCCAATTCTTGTTGAATTAGATGGTGATGTTAATGGGC<br>ACAAATTTTCTGTCCGTGGAGAGGGTGAAGGTGATGCTACAAACGGAAAACTCACCCT<br>TAAATTTATTTGCACTACTGGAAAACTACCTGTTCCATGGCCAACACTTGTCACTACTCT<br>GACCTATGGTGTTCAATGCTTTTCCCGTTATCCGGATCACATGAAACGGCATGACTTTT<br>TCAAGAGTGCCATGCCCGAAGGTTATGTACAGGAACGCACTATATCTTTCAAAGATGAC<br>GGGACCTACAAGACGCGTGCTGAAGTCAAGTTTGAAGGTGATACCCTTGTTAATCGTAT<br>CGAGTTAAAAGGTATTGATTTTAAAGAAGATGGAAACATTCTCGGACACAAACTCGAGT<br>ACAACTTTAACTCACACAATGTATACATCACGGCAGACAAACAAAAGAATGGAATCAAA<br>GCTAACTTCAAAATTCGCCACAACGTTGAAGATGGTTCCGTTCAACTAGCAGACCATTA<br>TCAACAAAATACTCCAATTGGCGATGGCCCTGTCCTTTTACCAGACAACCATTACCTGT<br>CGACACAATCTGTCCTTTCGAAAGATCCCAACGAAAAGCGTGACCACATGGTCCTTCTT<br>GAGTTTGTAACTGCTGCTGGGATTACACATGGCATGGATGAGCTCTACAAATAGTTAAG<br>TTTTCTCCTCGGTAGCTGACCGAGGGACCCCCGTAAACGGGGTGGGTGTGCTCGAAA<br>GAGCACGGGTGCGAAAGCGGTCCGGCTCCACCGAAAGGTGGGCGGGCTTCGGCCCA<br>GGGACCTCCCCCTAAAGAGAGGACCCGGGATTCTCCCGATTTGGTAACTAGCTGCTTG<br>GCTAGTTACCACCCTATAGTGAGTCGTATTAAGCTGTTTCCTGTGTGAAATTGTTATCC<br>GCTCACAATTCCACACAACATACGAGCCGGAAGCATAAAGTGTAAAGCCTGGGGTGCC<br>TAATGAGTGAGCTAACTCACATTAATTGCGTTGCGCTCACTGCCCGCTTTCCAGTCGGG<br>AAACCTGTCGTGCCAGCTGCATTAATGAATCGGCCAACGCGCGGGGAGAGGCGGTTT<br>GCGTATTGGGCGCTCTTCCGCTTCCTCGCTCACTGACTCGCTGCGCTCGGTCGTTCGG<br>CTGCGGCGAGCGGTATCAGCTCACTCAAAGGCGGTAATACGGTTATCCACAGAATCAG<br>GGGATAACGCAGGAAAGAACATGTGAGCAAAAGGCCAGCAAAAGGCCAGGAACCGTA<br>AAAAGGCCGCGTTGCTGGCGTTTTTCCATAGGCTCCGCCCCCCTGACGAGCATCACAA<br>AAATCGACGCTCAAGTCAGAGGTGGCGAAACCCGACAGGACTATAAAGATACCAGGCG<br>TTTCCCCCTGGAAGCTCCCTCGTGCGCTCTCCTGTTCCGACCCTGCCGCTTACCGGAT<br>ACCTGTCCGCCTTTCTCCCTTCGGGAAGCGTGGCGCTTTCTCATAGCTCACGCTGTAG<br>GTATCTCAGTTCGGTGTAGGTCGTTCGCTCCAAGCTGGGCTGTGTGCACGAACCCCCC<br>GTTCAGCCCGACCGCTGCGCCTTATCCGGTAACTATCGTCTTGAGTCCAACCCGGTAA<br>GACACGACTTATCGCCACTGGCAGCAGCCACTGGTAACAGGATTAGCAGAGCGAGGT<br>ATGTAGGCGGTGCTACAGAGTTCTTGAAGTGGTGGCCTAACTACGGCTACACTAGAAG<br>AACAGTATTTGGTATCTGCGCTCTGCTGAAGCCAGTTACCTTCGGAAAAAGAGTTGGTA<br>GCTCTTGATCCGGCAAACAAACCACCGCTGGTAGCGGTGGTTTTTTTGTTTGCAAGCA<br>GCAGATTACGCGCAGAAAAAAAGGATCTCAAGAAGATCCTTTGATCTTTTCTACGGGGT<br>CTGACGCTCAGTGGAACGAAAACTCACGTTAAGGGATTTTGGTCATGAGATTATCA	Forward:<br>5' GAAATAATACGACTCACTATAGGG TGGGACCCCTTTC 3'<br>Reverse:<br>5' GGTCAGCTACCGAGGAG 3'
pUCIDT_MS2_F30Bro	TCGCGCGTTTCGGTGATGACGGTGAAAACCTCTGACACATGCAGCTCCCGGAGACGG<br>TCACAGCTTGTCTGTAAGCGGATGCCGGGAGCAGACAAGCCCGTCAGGGCGCGTCAG<br>CGGGTGTTGGCGGGTGTCGGGGCTGGCTTAACTATGCGGCATCAGAGCAGATTGTAC<br>TGAGAGTGCACCAAATGCGGTGTGAAATACCGCACAGATGCGTAAGGAGAAAATACCG<br>CATCAGGCGCCATTCGCCATTCAGGCTGCGCAACTGTTGGGAAGGGCGATCGGTGCG<br>GGCCTCATCGCTATTACGCCAGCTGGCGAAAGGGGGATGTGCTGCAAGGCGATTAAG<br>TTGGGTAACGCCAGGGTTTTCCCAGTCACGACGTTGTAAAACGACGGCCAGTGCAACG<br>CGATGACGATGGATAGCGATTCATCGATGAGCTGACCCGATCGCCGCCGCCGGAGGG<br>TTGCGTTTGAGACGGGCGACAGATGGGTGGGACCCCTTTCGGGGTCCTGCTCAACTT<br>CCTGTCGAGCTAATGCCATTTTTAATGTCTTTAGCGAGACGCTACCATGGCTATCGCTG<br>TAGGTAGCCGGAATTCCATTCCTAGGAGGTTTTTGCCATGTGTATGTGGGAGACGGTC<br>GGGTCCAGATATTCGTATCTGTCGAGTAGAGTGTGGGCTCCCACATACTCTGATGATC	Forward:<br>5' GAAATAATACGACTCACTATAGGG TGGGACCCCTTTC 3'<br>Reverse:<br>5' GGTCAGCTACCGAGGAG 3'

	CTTCGGGATCATTCATGGCAACTCCTCGGTAGCTGACCGAGGGACCCCCGTAAACGG<br>GGTGGGTGTGCTCGAAAGAGCACGGGTGCGAAAGCGGTCCGGCTCCACCGAAAGGT<br>GGGCGGGCTTCGGCCCAGGGACCTCCCCCTAAAGAGAGGACCCGGGATTCTCCCGAT<br>TTGGTAACTAGCTGCTTGGCTAGTTACCACCCATCAGTTCTGGACCAGCGAGCTGTGC<br>TGCGACTCGTGGCGTAATCATGGTCATAGCTGTTTCCTGTGTGAAATTGTTATCCGCTC<br>ACAATTCCACACAACATACGAGCCGGAAGCATAAAGTGTAAAGCCTGGGGTGCCTAAT<br>GAGTGAGCTAACTCACATTAATTGCGTTGCGCTCACTGCCCGCTTTCCAGTCGGGAAA<br>CCTGTCGTGCCAGCTGCATTAATGAATCGGCCAACGCGCGGGGAGAGGCGGTTTGCG<br>TATTGGGCGCTCTTCCGCTTCCTCGCTCACTGACTCGCTGCGCTCGGTCGTTCGGCTG<br>CGGCGAGCGGTATCAGCTCACTCAAAGGCGGTAATACGGTTATCCACAGAATCAGGG<br>GATAACGCAGGAAAGAACATGTGAGCAAAAGGCCAGCAAAAGGCCAGGAACCGTAAAA<br>AGGCCGCGTTGCTGGCGTTTTTCCATAGGCTCCGCCCCCCTGACGAGCATCACAAAAA<br>TCGACGCTCAAGTCAGAGGTGGCGAAACCCGACAGGACTATAAAGATACCAGGCGTTT<br>CCCCCTGGAAGCTCCCTCGTGCGCTCTCCTGTTCCGACCCTGTCGCTTACCGGATACC<br>TGTCCGCCTTTCTCCCTTCGGGAAGCGTGGCGCTTTCTCATAGCTCACGCTGTAGGTA<br>TCTCAGTTCGGTGTAGGTCGTTCGCTCCAAGCTGGGCTGTGTGCACGAACCCCCCGTT<br>CAGCCCGACCGCTGCGCCTTATCCGGTAACTATCGTCTTGAGTCCAACCCGGTAAGAC<br>ACGACTTATCGCCACTGGCAGCAGCCACTGGTAACAGGATTAGCAGAGCGAGGTATGT<br>AGGCGGTGCTACAGAGTTCTTGAAGTGGTGGCCTAACTACGGCTACACTAGAAGAACA<br>GTATTTGGTATCTGCGCTCTGCTGAAGCCAGTTACCTTCGGAAAAAGAGTTGGTAGCTC<br>TTGATCCGGCAAACAAACCACCGCTGGTAGCGGTGGTTTTTTTGTTTGCAAGCAGCAG<br>ATTACGCGCAGAAAAAAAGGATCTCAAGAAGATCCTTTGATCTTTTCTACGGGGTCTGA<br>CGCTCAGTGGAACGAAAACTCACGTTAAGGGATTTTGGTCATGAGATTATCAAAAAGGA<br>TCTTCACCTAGATCCTTTTAAATTAAAAATGAAGTTTTAAATCAATCTAAAGTATATATGA<br>GTAAACTTGGTCTGACAGTTACCAATGCTTAATCAGTGAGGCACCTATCTCAGCGATCT<br>GTCTATTTCGTTCATCCATAGTTGCCTGACTCCCCGTCGTGTAGATAACTACGATACGG<br>GAGGGCTTACCATCTGGCCCCAGTGCTGCAATGATACCGCGAGACCCACGCTCACCG<br>GCTCCAGATTTATCAGCAATAAACCAGCCAGCCGGAAGGGCCGAGCGCAGAAGTGGT<br>CCTGCAACTTTATCCGCCTCCATCCAGTCTATTAATTGTTGCCGGGAAGCTAGAGTAAG<br>TAGTTCGCCAGTTAATAGTTTGCGCAACGTTGTTGCCATTGCTACAGGCATCGTGGTGT<br>CACGCTCGTCGTTTGGTATGGCTTCATTCAGCTCCGGTTCCCAACGATCAAGGCGAGT<br>TACATGATCCCCCATGTTGTGCAAAAAAGCGGTTAGCTCCTTCGGTCCTCCGATCGTTG<br>TCAGAAGTAAGTTGGCCGCAGTGTTATCACTCATGGTTATGGCAGCACTGCATAATTCT<br>CTTACTGTCATGCCATCCGTAAGATGCTTTTCTGTGACTGGTGAGTACTCAACCAAGTC<br>ATTCTGAGAATAGTGTATGCGGCGACCGAGTTGCTCTTGCCCGGCGTCAATACGGGAT<br>AATACCGCGCCACATAGCAGAACTTTAAAAGTGCTCATCATTGGAAAACGTTCTTCGGG<br>GCGAAAACTCTCAAGGATCTTACCGCTGTTGAGATCCAGTTCGATGTAACCCACTCGTG<br>CACCCAACTGATCTTCAGCATCTTTTACTTTCACCAGCGTTTCTGGGTGAGCAAAAACA<br>GGAAGGCAAAATGCCGCAAAAAAGGGAATAAGGGCGACACGGAAATGTTGAATACTCA<br>TACTCTACCTTTTTCAATATTATTGAAGCATTTATCAGGGTTATTGTCTCATGAGCGGAT<br>ACATATTTGAATGTATTTAGAAAAATAAACAAATAGGGGTTCCGCGCACATTTCCCCGAA<br>AAGTGCCACCTGACGTCTAAGAAACCATTATTATCATGACATTAACCTATAAAAATAGGC<br>GTATCACGAGGCCCTTTCGTC	

Table S2. DNA plasmid and primer sequences. Shown are the plasmid and primer sequences used to obtain a linear PCR fragment coding for sfGFP or F30-Broccoli. Highlighted in colors are the coding sequence of sfGFP or F30-Broccoli (green), forward and reverse primers (cyan) and the T7 promoter sequence (gray).

Movie S1. sfGFP expression recovery inside a thermophoretic chamber subjected to a temperature difference. Shown is a representative example of a 0.33x concentrated TX-TL reaction incubated for 16 h inside a thermophoretic chamber incubated within a 27-40 °C temperature difference. Over time, sfGFP signal starts appearing at the bottom fractions of the chamber. Scale bar = 1 mm.

Movie S2. Unfunctional TX-TL reaction inside a thermophoretic chamber isothermally incubated at 37 °C. Shown is a representative example of a 0.33x concentrated TX-TL reaction incubated for 16 h inside a thermophoretic chamber incubated at 37 °C. sfGFP signal was not detected over the entire course of the experiment. Scale bar = 1 mm.

SI References

1. Weise, L. I., Heymann, M., Mayr, V. & Mutschler, H. Cell-free expression of RNA encoded genes using MS2 replicase. *Nucleic Acids Research* **47**, 10956–10967 (2019).
2. Matreux, T. *et al.* Heat flows in rock cracks naturally optimize salt compositions for ribozymes. *Nat. Chem.* **13**, 1038–1045 (2021).
3. Cohen, S. A. & Michaud, D. P. Synthesis of a Fluorescent Derivatizing Reagent, 6-Aminoquinolyl-N-Hydroxysuccinimidyl Carbamate, and Its Application for the Analysis of Hydrolysate Amino Acids via High-Performance Liquid Chromatography. *Analytical Biochemistry* **211**, 279–287 (1993).
4. Reichl, M., Herzog, M., Götz, A. & Braun, D. Why Charged Molecules Move Across a Temperature Gradient: The Role of Electric Fields. *Physical Review Letters* **112**, 198101 (2014).
5. Keil, L. M. R., Möller, F. M., Kieß, M., Kudella, P. W. & Mast, C. B. Proton gradients and pH oscillations emerge from heat flow at the microscale. *Nat Commun* **8**, 1897 (2017).
6. Bowen, W. J. & Martin, H. L. The diffusion of adenosine triphosphate through aqueous solutions. *Archives of Biochemistry and Biophysics* **107**, 30–36 (1964).
7. Anand, V. S. & Patel, S. S. Transient State Kinetics of Transcription Elongation by T7 RNA Polymerase * ♦. *Journal of Biological Chemistry* **281**, 35677–35685 (2006).
8. Lavickova, B. & Maerkl, S. J. A Simple, Robust, and Low-Cost Method To Produce the PURE Cell-Free System. *ACS Synth. Biol.* **8**, 455–462 (2019).
